# A Hybrid Black-Winged Kite Algorithm with PSO and Differential Mutation for Superior Global Optimization and Engineering Applications

**DOI:** 10.3390/biomimetics10040236

**Published:** 2025-04-11

**Authors:** Xuemei Zhu, Jinsi Zhang, Chaochuan Jia, Yu Liu, Maosheng Fu

**Affiliations:** 1 Experimental Training Teaching Management Department, West Anhui University, Yu’an District, Lu’an 237012, China; 14000012@wxc.edu.cn; 2 School of Electrical and Optoelectronic Engineering, West Anhui University, Yu’an District, Lu’an 237012, China; 3 School of Electronics and Information Engineering, West Anhui University, Yu’an District, Lu’an 237012, China; 03000076@wxc.edu.cn (C.J.); liuyui15005510@163.com (Y.L.); fums@wxc.edu.cn (M.F.)

**Keywords:** hybrid algorithm, Random-Elite Difference Mutation, global optimization, benchmark functions, engineering design

## Abstract

This study addresses the premature convergence issue of the Black-Winged Kite Algorithm (BKA) in high-dimensional optimization problems by proposing an enhanced hybrid algorithm (BKAPI). First of all, BKA provides dynamic global exploration through its hovering and dive attack strategies, while Particle Swarm Optimization (PSO) enhances local exploitation via its velocity-based search mechanism. Then, PSO enables efficient local refinement, and Differential Evolution (DE) introduces a differential mutation strategy to maintain population diversity and prevent premature convergence. Finally, the integration ensures a balanced exploration–exploitation trade-off, overcoming BKA’s sensitivity to parameter settings and insufficient local search capabilities. By combining these mechanisms, BKAPI achieves a robust balance, significantly improving convergence speed and computational accuracy. To validate its effectiveness, the performance of the enhanced hybrid algorithm is rigorously evaluated against seven other intelligent optimization algorithms using the CEC 2017 and CEC 2022 benchmark test functions. Experimental results demonstrate that the proposed integrated strategy surpasses other advanced algorithms, highlighting its superiority and strong application potential. Additionally, the algorithm’s practical utility is further confirmed through its successful application to three real-world engineering problems: welding beam design, the Himmelblau function, and visible light positioning, underscoring the effectiveness and versatility of the proposed approach.

## 1. Introduction

Intelligent optimization algorithms, inspired by natural phenomena and biological behaviors, are increasingly being used to optimize various tasks, including design parameters, scheduling, resource allocation, path planning, control systems, and process optimization [1,2,3,4]. These algorithms are particularly effective for addressing complex challenges, such as improving performance, enhancing efficiency, and reducing costs across multiple industries [5,6,7] when compared to conventional methods like the Conjugate Gradient and Newton methods [8,9,10]. Meta-heuristic algorithms [11,12,13], a category of computational methods that mimic natural phenomena, are designed to find global optimal solutions in intricate search spaces, making them highly suitable for tackling high-dimensional, nonlinear, and multi-modal problems [14]. Examples of these algorithms include Genetic Algorithms (simulating biological evolution) [15], Particle Swarm Optimization (mimicking the collective behavior of birds or fish) [16], Gray Wolf Optimization (simulating the hunting strategies of gray wolves) [17], Differential Evolution (group-based optimization through cooperation and competition) [18], simulated annealing (mimicking the annealing process) [19], cuckoo search (based on bird breeding behaviors) [20], and Ant Colony Optimization (based on ants’ foraging paths) [21]. An adaptive Differential Evolution algorithm (AdapDE) with adaptive operator selection and parameter control aims at optimizing the input weights and hidden biases of extreme learning machines (ELMs), enhancing performance through improved adaptive operator selection mechanisms and parameter adjustment methods [22]. A multi-objective evolutionary algorithm based on decomposition (MOEA/D) combined with an adaptive operator selection strategy called FRRMAB, demonstrating superior performance on various test instances and emphasizing its effectiveness in balancing exploration and exploitation [23]. Each of these algorithms leverages unique aspects of nature to explore and exploit the solution space efficiently.

Despite their strengths, individual meta-heuristic algorithms often face challenges when applied to highly complex or high-dimensional problems. For example, the GA excels in global search but struggles with local optimization, while PSO converges quickly but may get trapped in local optimal solutions [24]. Hybrid intelligent optimization algorithms have emerged as a promising solution to overcome these limitations. Hybrid approaches achieve superior optimization performance compared to individual methods by combining the strengths of multiple algorithms. Examples include the fusion of the GA and PSO [25], DE and SA [26], and ABC and GWO [27]. These hybrid algorithms leverage the complementary strengths of their constituent methods, enhancing both global and local search efficiency and addressing the limitations of individual algorithms [28,29,30,31,32].

The Black-Winged Kite Algorithm (BKA) [33], one of the more recent optimization techniques, has attracted considerable interest due to its distinctive inspiration derived from the hunting strategies of the black-winged kite. The BKA emulates two primary search strategies, local search (hovering) and global search (dive attack), effectively balancing exploration and exploitation to circumvent local optima while efficiently locating the global optimal solution. However, the BKA faces significant challenges when applied to high-dimensional or complex nonlinear problems. Its tendency to converge prematurely, known as “precocious convergence”, reduces its effectiveness in navigating complex landscapes with multiple local optima [34]. Additionally, the BKA’s performance is highly sensitive to parameter settings, and its local search capabilities are often insufficient for high-dimensional problems, leading to increased computational costs and reduced solution quality [35].

To overcome these limitations, researchers have introduced a range of improvements to the BKA. Rasooli et al. advanced a clustering solution based on the BKA for data analysis and machine learning [36], while Zhang et al. combined the black-winged kite with another similar bird, the osprey, to solve optimization problems [37]. Xue et al. proposed a multi-strategic integrated model combining the BKA and artificial rabbit optimization [38], and Zhao et al. enhanced the BKA using chaotic mapping and confrontation learning to enhance its capability to avoid becoming trapped in local optima [39]. Fu et al. integrated the BKA with deep hybrid nuclear limit learning machines to enhance prediction accuracy for battery state-of-health [40]. These improvements highlight the potential of the BKA but also underscore the need for further enhancements to address its constraints in high-dimensional and sophisticated optimization tasks.

The hybridization of metaheuristic algorithms has been the subject of considerable academic attention, with various strategies combining different algorithms to achieve better performance [41]. For instance, hybrid algorithms like GA-PSO (Genetic Algorithm with PSO) [15] and DE-ABC (Differential Evolution with Artificial Bee Colony) [18] have demonstrated improved performance in specific contexts. Several novel bio-inspired algorithms have been proposed, each offering unique mechanisms for balancing exploration and exploitation. For instance, the Subtraction-Average-Based Optimizer (SABO) [42] introduces a novel subtraction-average mechanism to enhance population diversity and avoid premature convergence. Similarly, the Mantis Search Algorithm (MSA) [43] mimics the hunting behavior of mantises, incorporating a dynamic balance between exploration and exploitation through a unique prey–predator interaction model. The Kepler Algorithm (KA) [44] draws inspiration from Kepler’s laws of planetary motion, utilizing orbital dynamics to guide the search process. Additionally, the Improved Bio-Inspired Material Generation Algorithm (IMGMA) [45] enhances the original Material Generation Algorithm by incorporating adaptive mechanisms to improve convergence speed and solution accuracy. However, these hybrid algorithms often face challenges in consistently balancing exploration and exploitation across different types of problems. In contrast, the proposed BKAPI integrates the BKA’s dynamic exploration with PSO’s efficient exploitation, creating a more versatile and robust optimization framework. Additionally, the inclusion of the Random-Elite Difference Mutation strategy provides a unique mechanism for maintaining diversity, which is often lacking in other hybrid approaches. This makes the BKAPI particularly effective for solving optimization problems that are complex and multimodal.

This paper seeks to address the above research gap by proposing a novel hybrid algorithm, BKAPI, which integrates the superior aspects of the BKA in contrast with alternative optimization approaches to address its problems. The proposed algorithm integrates mechanisms to improve performance, thereby reducing susceptibility to premature settling and refining solution performance. The remainder of this paper is structured as follows: Section 2 clarifies the fundamental aspects of the BKA through a flow diagram and calculation formulas, describes the proposed BKAPI, and provides its pseudo-code. Section 3 analyzes the performance outcomes of eight distinct algorithms, including the BKAPI. Section 4 applies the algorithm to three engineering cases. Finally, this paper concludes with a summary and a discussion on the potential directions for future work.

## 2. Black-Winged Kite Algorithm

The Black-Winged Kite Algorithm (BKA) is a metaheuristic optimization algorithm based on the predation behavior of the black-winged kite, simulating its hovering foraging (local search) and dive attack (global search) behaviors to find the optimal solution in the solution space [33]. This equilibrium between local and global exploration is essential for enabling the algorithm to identify the global optimum in challenging optimization problems. Additionally, the BKA incorporates migration behavior, which introduces randomness based on the Cauchy distribution [33].

### 2.1. Population Initialization

In the BKA, the initialization matrix of Pop × Dim is generated: Pop is the population size, and Dim is the dimension of the problem. The population matrix BK is defined as follows:

(1) $$BK=\left[\begin{array}{cccc} BK_{1,1} & BK_{1,2} & \cdots & BK_{1,\dim}\\ BK_{2,1} & BK_{2,2} & \cdots & BK_{2,\dim}\\ \vdots & \vdots & \ddots & \vdots \\ BK_{\mathrm{pop},1} & BK_{\mathrm{pop},2} & \cdots & BK_{\mathrm{pop},\dim} \end{array}\right],$$

### 2.2. Attacking Behavior

Local exploration: If p<r, indicating global exploration, the formula is as follows:

(2) $$\;k_{t+1}^{i,j}=k_{t}^{i,j}+n\;\cdot \;\left(1+\sin\left(r\right)\right)\;\cdot \;k_{t}^{i,j}\;$$

Here, $k_{t}^{i,j}$ represents the current position of the black-winged kite individual labeled *i* and $k_{t+1}^{i,j}$ represents the new position after the update. Local exploration nearby resolves spaces near the current position.

Local exploration: If p>r, indicating partial exploration, the formula is as follows:

(3) $$\;k_{t+1}^{i,j}=k_{t}^{i,j}+n\;\cdot \;\left(2r-1\right)\;\cdot \;k_{t}^{i,j}\;$$

where *r* is a random number and *n* is the adaptive step size:

(4) $$\;n=0.05\;\cdot \;\exp\left(-2{\left(\frac{t}{T}\right)}^{2}\right)\;$$

where $k_{t}^{i,j}$ represents the current position of the black-winged kite individual labeled *i* and $k_{t+1}^{i,j}$ represents the newer position after the update. Global exploration is achieved by introducing larger random disturbances to explore a larger space.

### 2.3. Migration Behavior

The leader steps down and joins the migrating population if the fitness of the current population is lower than that of a randomly selected individual but retains its position and continues to guide the population if its fitness is higher.

A reference individual ri is generated randomly from the population. Reference fitness is computed as $F_{ri}=f\left(k_{t}^{ri,j}\right)$

If $F_{i}<F_{ri}$, the leader steps down and the formula is as follows:

(5) $$\;k_{t+1}^{i,j}=k_{t}^{i,j}+C\left(0,1\right)\;\cdot \;\left(k_{t}^{i,j}-L_{t}^{j}\right)\;$$

If $F_{i}>F_{ri}$, the leader retains its position and the formula is as follows:

(6) $$\;k_{t+1}^{i,j}=k_{t}^{i,j}+C\left(0,1\right)\;\cdot \;\left(L_{t}^{j}-m\;\cdot \;k_{t}^{i,j}\right)\;$$

where $L_{t}^{j}$ represents the leader of the black-winged kites in the $j^{th}$ dimension of the $t^{th}$ iteration so far, $k_{t}^{i,j}$ represents the current position of the black-winged kite individual labeled *i*, and $k_{t+1}^{i,j}$ represents the newer position after the update, which is $m=2\;\cdot \;sin(r+\frac{\pi }{2})$ and C(0,1)=tan((ori_value−0.5)·π).

## 3. Enhanced Black-Winged Kite Optimization

While the Black-Winged Kite Algorithm (BKA) presents some unique advantages, it may experience difficulties when facing problems with highly nonlinear or multiple local optimal solutions. Specifically, the BKA sometimes converges to suboptimal solutions too quickly, meaning that it may stop searching without fully exploring all potential solutions. This premature convergence phenomenon is particularly evident in high-dimensional and complex problems, which may result in the need for larger population sizes and more iterations to achieve satisfactory results, thereby increasing computational costs. Furthermore, the BKA is very sensitive to parameter selection, especially when dealing with high-dimensional problems. If the parameters are not set properly, the algorithm may not be able to search effectively and may even converge too prematurely. Moreover, as the problem dimension increases, the BKA’s local search ability appears to be relatively insufficient, and it struggles to escape from the local optimal solution and find the global optimal solution. It is possible to integrate the PSO mechanism and random elite differential mutation strategies into the basic BKA to enhance the BKA’s ability to tackle high-dimensional and complex nonlinear problems. This hybrid approach leverages the strengths of both methods to overcome stagnation and improve convergence during the BKA search process.

### 3.1. PSO

The Particle Swarm Intelligent Optimization Algorithm (PSO) is an optimization algorithm based on group behavior. By simulating the social behavior of birds or fishes, individuals follow the current optimal solution in the solution space and constantly update their own position to find the global optimal solution. The PSO component updates the particles’ velocities and positions using cognitive and social components.

Velocity Update:

(7) $$\;V_{t+1}^{i,j}=w\;\cdot \;V_{t}^{i,j}+c_{1}\;\cdot \;rand\;\cdot \;\left(k_{t+1}^{i,j}-k_{t}^{i,j}\right)+c_{2}\;\cdot \;rand\;\cdot \;\left(L_{t}^{j}-k_{t}^{i,j}\right)\;$$

(8) $$\;k_{t+1}^{i,j}=k_{t}^{i,j}+V_{t+1}^{i,j}$$

where *w* is the inertia weight controlling exploration and exploitation. $c_{1}$ and $c_{2}$ are learning factors for personal and global components. $k_{t+1}^{i,j}$ is the particle’s best-known position. $L_{t}^{j}$ is the global best position across all particles.

In the traditional BKA algorithm, the search process primarily focuses on local exploration, which, while efficient within a specific area of the solution space, tends to get stuck in local minima. The global search mechanism of PSO addresses this shortcoming by introducing a broader exploration strategy; this enables the algorithm to explore a broader spectrum of potential solutions. PSO achieves this by updating particle positions based on two primary influences, namely the global best (collective experience) and the personal best (individual experience), leading to more thorough global exploration across the solution space [46].

### 3.2. Random-Elite Difference Mutation

Random-Elite Difference Mutation is a probability-based variant strategy that guides individuals to evolve towards better solutions by combining global optimal solutions and randomly selected individual differences while introducing randomness to enhance population diversity. It works as follows:

(1) Random Selection for Mutation: A value for rand is randomly generated within the range [0, 1]. If rand > *p*, the mutation operation is triggered. Otherwise, the individual solution remains unchanged. This probabilistic approach ensures that not every individual undergoes mutation, promoting exploration and stability.
(2) Mutation Formula: When mutation occurs, the new solution is computed using the following formula:
  (9) $$\;k_{t+1}^{i,j}=k_{t}^{i,j}+F\;\cdot \;\left(L_{t}^{,j}-k_{t}^{i,j}\right)+F\;\cdot \;(BK^{i,j}\left(r_{1}\left(np,:\right)\right)-(BK^{i,j}\left(r_{2}\left(np,:\right)\right)\;$$
  $k_{t}^{i,j}$ is the current position vector of an individual.
  $L_{t}^{,j}$ represents the global best solution found so far.
  $BK^{i,j}$ is the matrix of all individuals in the population.
  *F* is a scaling factor that controls the magnitude of the mutation.
  r(np) is used to randomly select two different population members for the second mutation term.

The formula consists of two parts:

Global Best Influence: $F\;\cdot \;(L_{t}^{,j}-k_{t}^{i,j})$ represents the difference between the global best solution and the current individual. By scaling this difference by the factor *F*, the individual is attracted towards the best solution found so far.

Random Population Influence: $F\;\cdot \;(BK^{i,j}\left(r_{1}\left(np,:\right)\right)-(BK^{i,j}\left(r_{2}\left(np,:\right)\right)$ introduces a random component by selecting two different individuals from the population. This term introduces more diversity by altering the individual based on the difference between two randomly chosen solutions.

Additional mutations using a custom operator (mutations) are applied to improve diversity [47]. In the BKA section, the differential mutation strategy in the mutation function is called the mutant operation to the new position. The mutation mechanism can introduce new solutions, enhance the diversity of populations, and prevent premature convergence to a local optimum. Mutation operations help the algorithm to maintain a better exploration ability in complex issues. To increase population diversity, a Random-Elite Difference Mutation strategy is added to the algorithm. The mutation operation is governed by a probability *p*.

The Particle Swarm Optimization (PSO) mechanism was selected due to its established ability to search globally and converge effectively. The higher speed rules of PSO integrate both cognitive (personal best) and social (world best) components, allowing the algorithm to effectively explore search spaces while leveraging high-quality solutions. This feature makes PSO particularly suitable for accelerated convergence in complex optimization problems. To address the limitations of traditional mutation operators that usually lack direction, random elite differential mutation strategies have been incorporated. This strategy introduces controlled diversity by utilizing the differences between elite and random solutions to investigate new areas of the search space without sacrificing convergence speed. By integrating these strategies, global search capabilities are improved, and convergence is expedited, making the algorithm highly effective in addressing complex optimization challenges. The synergy between these mechanisms improves robustness and adaptability, ensuring outstanding performance in complex situations. The computational complexity of BKAPI is analyzed to measure its runtime consumption; the component is as follows: initialization O(N), position update of the search agent O(Tmax × N × Dim), and fitness evaluation O(Tmax × N), where N stands for the population size, Dim represents the dimension, and Tmax is the maximum iterations. Consequently, the total computational complexity of BKAPI is O(BKAPI) = O(initialization) + O(position update) + O(fitness evaluation).

**Figure 1:**
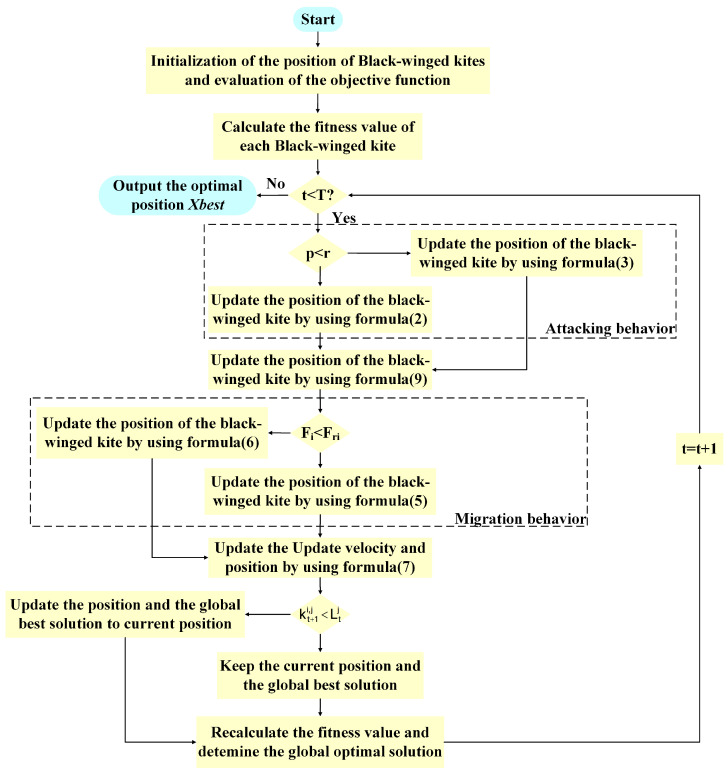
The flow chart of BKAPI.

In the hybrid improved BKA based on the PSO algorithm, PSO’s global search is alternated with BKA’s local search, allowing the BKAPI to first explore the global search space and then focus on refining solutions through local searches.The pseudocode of BKAPI (Algorithm 1) is provided, offering a clear description of the execution process. The flow chart of the BKAPI is shown in Figure 1. Specifically, the hybrid strategy uses the PSO algorithm for a global search during the early iterations, where w represents the inertia weight, the individual learning factor, and the global learning factor guiding particles toward the best solutions. In contrast, the BKA focuses on more fine-tuned local exploration, especially after promising global solutions are identified. By adding these two components together, the algorithm explores the search space with greater effectiveness, allowing individuals to evolve toward effective solutions while maintaining diversity.
**Algorithm 1** BKAPI algorithm**Require:** pop: Population size.  
dim
: Dimension of the problem.  
ub
, lb: Upper and lower bounds for each dimension. 
*T*: Maximum number of iterations.
 
fobj
: Objective function.
**Ensure:** Xbest: The best quasi-optimal solution obtained by BKAPI for a given optimization problem.
 
Fbest
: The fitness value of the best solution.
**Initialization phase:** Initialize the population positions, PSO velocities, personal best positions, global best position, and global best fitness.**for** t=1 to *T* **do**    **BKA Phase: Local Search (Attacking behavior)**    **if** p<r **then**        Update the position applying Equation (2).    **else if** p≥r **then**        Update the position applying Equation (3).    **end if**    **Differential Evolution**    Update the position applying Equation (9).    Evaluate the fitness objective function.    **Migration behavior**    Generate a reference individual ri randomly from the population.    Compute reference fitness $F_{ri}=f\left(k_{t}^{ri,j}\right)$.    **if** $F_{i}<F_{ri}$ **then**        Update the position applying Equation (5).    **else**        Update the position applying Equation (6).    **end if**    Evaluate the fitness objective function.    **PSO Phase: Global Search**    Update velocity and position using Equation (7).    **Boundary handling**    Ensure the position stays within bounds.    Evaluate the fitness objective function.    **Select the best individual**    **if** $k_{t+1}^{i,j}<L_{t}^{j}$ **then**        $Xbest=k_{t+1}^{i,j},Fbest=f\left(k_{t+1}^{i,j}\right)$    **else**        $Xbest=L_{t}^{j},Fbest=f\left(L_{t}^{j}\right)$    **end if**    **Update global best****end for****return** Best Fitness and Best Position.


### 3.3. Ablation Study of BKAPI

**Figure 2:**
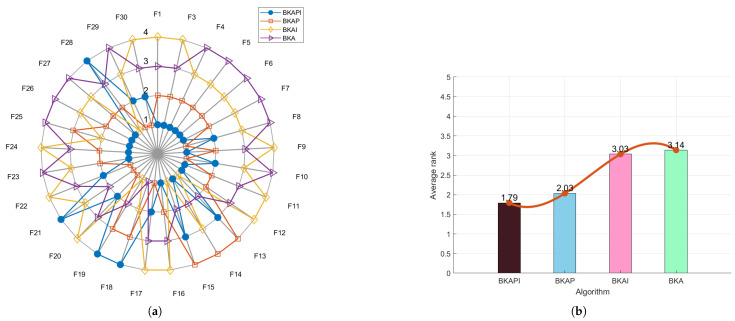
The comparison chart of the ablation study based on the average rank for CEC 2017. (**a**) The radar chart. (**b**) The average rank chart (the comparison chart of ablation study based on average rank for CEC 2017).

**Figure 3:**
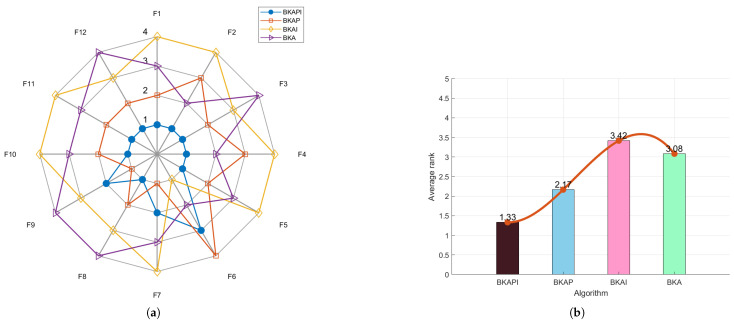
The comparison chart of the ablation study based on the average rank for CEC 2022. (**a**) The radar chart. (**b**) The average rank chart (the comparison chart of algorithm performance based on average rank for CEC 2022).

The ablation study of BKAPI involves analyzing its comparative performance with related algorithms, including hybrid Black-Winged Kite Algorithm based on particle swarm algorithm (BKAP), BKA, and improved Black-Winged Kite Algorithm based on mutation operations (BKAI), using the provided radar plots and ranking bar charts (Figure 2 for CEC2017 and Figure 3 for CEC2022). The radar plots depict BKAPI’s superior performance across multiple functions, with its position consistently closer to the center, indicating lower rankings and better stability. In contrast, BKA exhibits the widest spread, suggesting higher variance and weaker performance. The ranking bar charts further confirm BKAPI’s effectiveness. For the CEC 2017 dataset, BKAPI achieves the lowest average rank (1.79), followed by BKAP (2.03), BKAI (3.03), and BKA (3.14). Similarly, for the CEC 2022 dataset, BKAPI maintains the best rank (1.33), with BKAP at 2.17, BKA at 3.08, and BKAI at 3.42. The consistent trend across both datasets highlights BKAPI’s robustness in comparison to its alternatives.

The study also provides insight into the impact of individual components within BKAPI. The significant improvement from BKAP to BKAPI suggests that the additional mechanisms incorporated into BKAPI play a crucial role in optimizing performance. The relatively poor performance of BKA further underscores the importance of these enhancements, as its lack of improvements results in greater instability and lower rankings. The combination of radar plots and ranking metrics solidifies BKAPI’s dominance, demonstrating its efficiency and stability across diverse test functions. Overall, the ablation study confirms that BKAPI is the most effective algorithm among its variants, making it the optimal choice for solving complex optimization problems.

## 4. Experimental Analysis

Test sets were selected based on research requirements and algorithm characteristics to assess the improved Black-Winged Kite Algorithm (BKAPI). Classic functions were used for preliminary verification, while the CEC series was employed for in-depth evaluation. Following the principle that multiple test sets offer a thorough evaluation of the performance, CEC 2017 was selected to validate the effectiveness for complex continuous optimization problems, and CEC 2022 was selected to evaluate its performance in the latest complex optimization scenarios. These test functions are used to assess the performance of optimization algorithms across various complex problem scenarios; to ensure fairness, all algorithms were executed on the same computational platform under consistent conditions during the experiments.

### 4.1. Experimental Setting

Choosing appropriate comparison algorithms is critical to ensuring that the results of the experiments are both scientific and comparable. Effective selection helps to evaluate the new algorithm’s performance and offers insights for potential improvements [48]. The following criteria should be considered when selecting comparison algorithms. Classic Benchmark Algorithms: These are well-established algorithms that ensure the experimental results are widely accepted and comparable. Algorithms with similar mechanisms allow for an exhaustive study of the unique aspects of the new algorithm in comparison to others with similar features, including new algorithms. Incorporating the latest algorithms ensures that the results are timely and relevant to current optimization challenges. Specialized Algorithms for Specific Problems: These algorithms are selected to evaluate the new algorithm’s performance in specialized application scenarios, offering insights into its practical effectiveness. According to the above principles, eight algorithms were selected for comparison, namely the BKA, PSO, the Genetic Algorithm (GA) [25], the Cuckoo Optimization Algorithm (COA) [49], Beluga Whale Optimization (BWO) [50], Adaptive Optics (AO) [51], and the Starfish Optimization Algorithm (SFOA) [52], based on the test sets CEC 2017 and 2022. The experiments were equipped with an Intel^®^ Core Ultra 9 185H 2.30 GHz processor (Intel Corporation, Santa Clara, CA, USA) and a Windows 11 operating system.

The implementation of all algorithms was carried out using MATLAB 2024a, with a population size set to 30 and 300 iterations per run, and each algorithm was executed 30 times. The dimensionality of the problem was set to 10 and 100. The selection of these parameters aimed to find an equilibrium between computational efficiency, statistical validity, and algorithm performance. This configuration represents a common experimental design in optimization algorithm research, effectively assessing algorithmic performance while conserving computational resources. If higher precision or the handling of more complex problems is required, these parameters can be appropriately adjusted (e.g., by increasing the population size).

### 4.2. Results and Analysis of the Test Functions for CEC 2017 with Dimensions of 10 and 100

Categories of test functions (F1–F30) include unimodal functions with Gaussian noise (F1–F3) that can test an algorithm’s convergence ability in smooth, unimodal landscapes. Simple multimodal functions (F4–F10) can simulate scenarios with multiple local minima. Hybrid functions (F11–F20) can combine several basic functions into one, creating heterogeneous landscapes. Composition functions (F21–F30) can compound multiple multimodal functions into a single problem to simulate highly complex landscapes [41]. Std and avg represent the standard deviation and average, respectively, as shown in Table 1 and Table 2.

The BKAPI was evaluated against seven other intelligent optimization algorithms: BKA, PSO, GA, COA, BWO, AO, and SFOA. The BKAPI consistently excels in both average performance and stability (standard deviation) across a range of functions. It frequently achieves the lowest avg and std values, demonstrating both superior optimization and stability. For F1, the BKAPI achieves the best results with an avg value of 5.71 $\times \;10^{3}$. and a std value of 5.05 $\times \;10^{3}$, showing high optimization capability and stability. Other algorithms, like BWO and AO, show extremely high avg and std values, indicating poor performance. For F3, the BKAPI and PSO deliver a similar avg value of 3.00 $\times \;10^{2}$, but the BKAPI has a much smaller std value, 3.74 $\times \;10^{-11}$, demonstrating far better stability. The GA and COA show higher variability and worse fitness, with an avg value reaching 5.47 $\times \;10^{4}$; this indicates that the BKAPI exhibits the strongest convergence capability in smooth, unimodal landscapes. For functions F4 to F10, the BKAPI achieves both the lowest average values and the smallest standard deviation, as indicated in bold. BWO and AO exhibit significantly higher avg values and std values, reflecting inefficiency in tackling this problem. The analysis demonstrates that the BKAPI exhibits a robust overall optimization capability, effectively preventing premature convergence and avoiding local optima. The BKAPI can achieve the optimal solution within the search space, highlighting its efficiency in finding globally optimal solutions across various test functions. For F11–F20, while most algorithms perform poorly (e.g., avg value of 1.04 $\times \;10^{4}$ for AO of F11), the BKAPI maintains impressive avg values (1.14 $\times \;10^{3}$) and small std values (4.88 $\times \;10^{1}$) for F11. The BKAPI significantly outperforms the other algorithms with regard to F11, with an avg value of 2.16 $\times \;10^{4}$ and a std value of 1.62 $\times \;10^{4}$ of F18. The BKA follows closely, but algorithms like the GA demonstrate poor performance, which shows that the BKAPI features an extensive search range, high population diversity, and robust global exploration capabilities. Incorporating Random-Elite Difference Mutation enhances population diversity, effectively preventing premature convergence. This mechanism filters out the best search agents, thereby strengthening local search precision. The complementary advantages of these features ensure that the algorithm avoids search stagnation. For functions F21 to F30, the differences between the eight algorithms are not significant; however, overall, the BKAPI demonstrates the best performance. This indicates that the BKAPI is more capable of solving highly complex landscape problems compared to the BKA algorithm, which highlights one of the main limitations of the BKA.

The BKAPI has the lowest std values across most functions, highlighting its robustness and reliability. Algorithms like COA, BWO, and AO often exhibit high std values, reflecting inconsistency in optimization performance. PSO performs reasonably well in terms of std values but falls short compared to the BKAPI. The BKAPI generally surpasses the BKA in both avg values and std values, showcasing the advantage of the hybrid integration with enhanced global search and faster convergence. PSO occasionally matches the BKAPI in avg values (e.g., (F3)), but its higher std values suggest lower reliability. The BKAPI consistently outperforms the GA, COA, BWO, AO, and SFOA in both the average fitness and spread of results across nearly all functions. Its superior optimization performance and stability make the BKAPI highly effective for addressing diverse and complex problems in the CEC 2017 benchmark. Algorithms like PSO and the BKA are competitive in specific cases but lack the consistency of the BKAPI. The GA, COA, and AO show significant limitations, with high fitness values and variability across functions, especially for complex or high-dimensional problems.

The Wilcoxon rank-sum test is employed to assess if there is a significant statistical difference between two independent data sets. In this context, it was used to assess whether the performance of the BKAPI significantly differed from that of the other algorithms [53]. To analyze the table for values greater than 0.05 (5 $\times \;10^{-2}$), which is a commonly used threshold in statistics for determining the significance of results, we identified cases where the *p*-value exceeded this threshold, which are described in Table 3. A *p*-value greater than 5 $\times \;10^{-2}$ typically suggests that the difference is not statistically significant and may be attributed to random variation rather than a genuine effect. From the data, it is observed that the COA, BWO, AO, and SFOA frequently achieve the smallest *p*-value, at 1.73 $\times \;10^{-6}$, indicating that the BKAPI demonstrates statistically significant differences for the corresponding test functions. Conversely, PSO, BKA, and the SFOA display larger *p*-values for multiple functions, suggesting that their performance is occasionally similar. This similarity arises because the BKAPI builds upon and improves the features of the BKA and PSO. Additionally, the SFOA, as a newly proposed and effective algorithm, also shows competitive performance in certain cases, reflecting its potential alongside the BKAPI in achieving high optimization accuracy.

**Figure 4:**
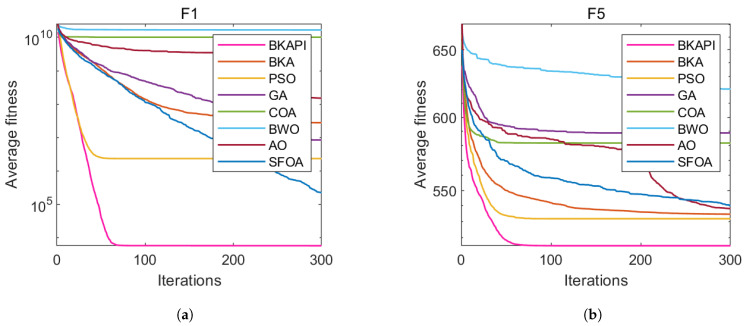

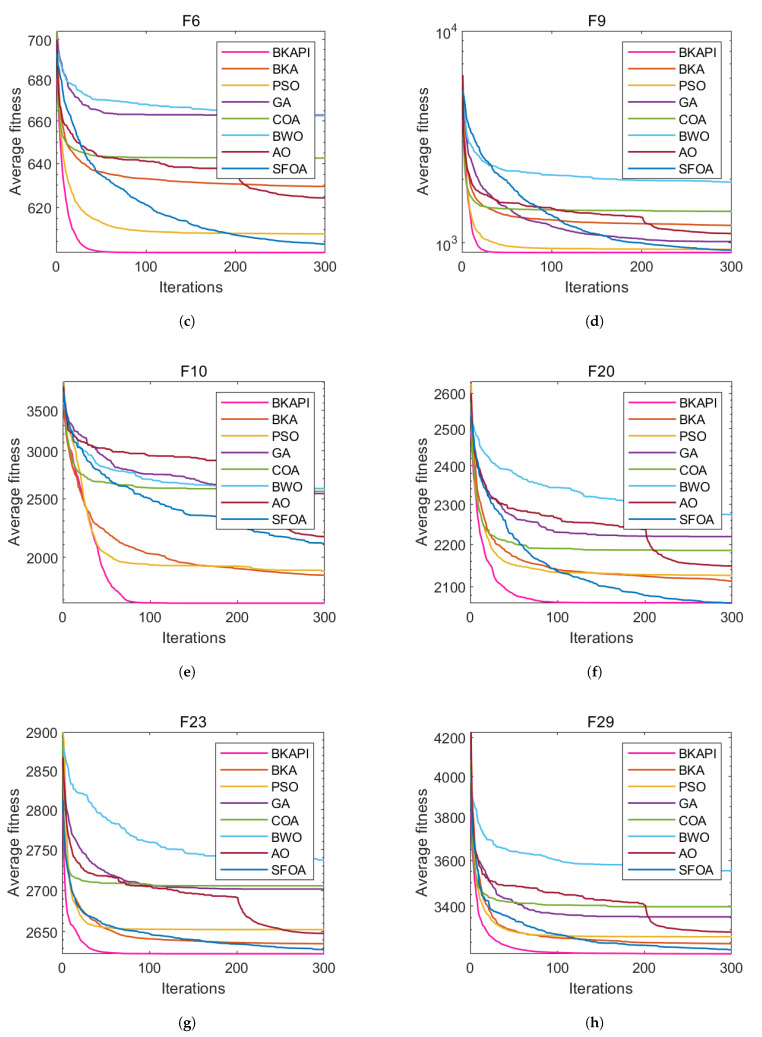
Parts of the convergence analysis of the BKAPI and competing algorithms on selected functions of the CEC 2017 benchmark with dimensions of 10. (**a**) f1, (**b**) f5, (**c**) f6, (**d**) f9, (**e**) f10, (**f**) f20, (**g**) f23, (**h**) f29.

**Figure 5:**
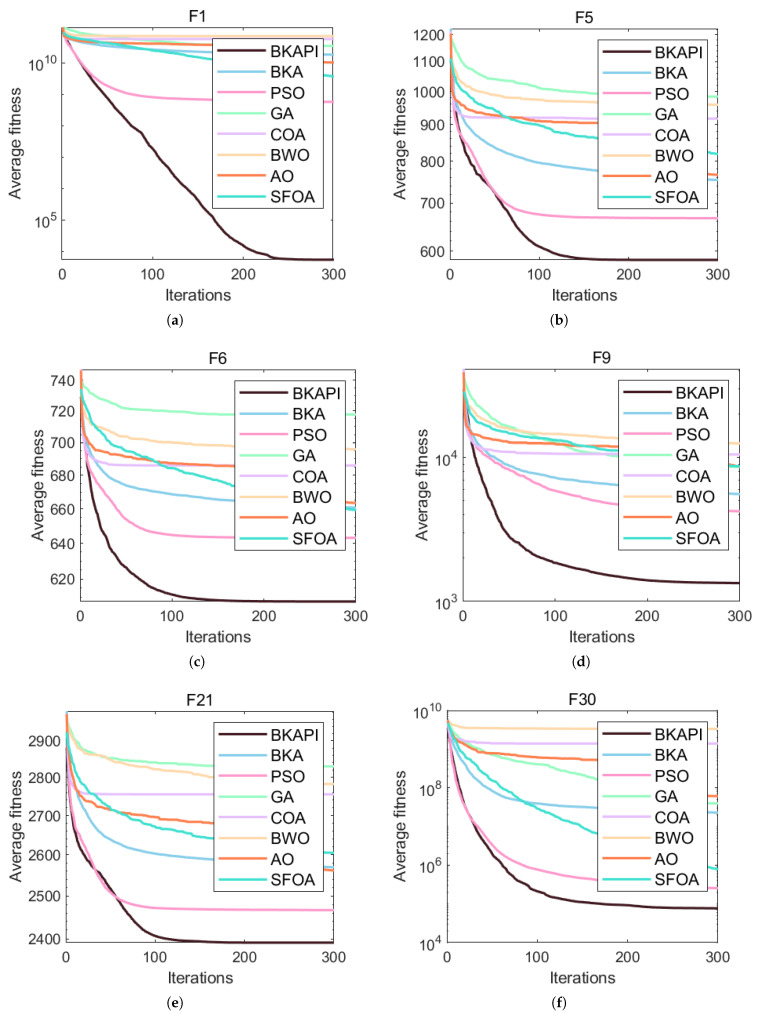
Parts of the convergence analysis of BKAPI and competing algorithms on selected functions of the CEC 2017 benchmark with dimensions of 100. (**a**) f1, (**b**) f5, (**c**) f6, (**d**) f9, (**e**) f21, (**f**) f30.

The images above present parts of the fitness convergence plots comparing various optimization algorithms across different benchmark objective functions of CEC 2017 with dimensions of 10 (Figure 4) and 100 (Figure 5). The number of iterations is represented on the x-axis, while the average fitness values are shown on the y-axis, either on a logarithmic or linear scale, depending on the function. The algorithm’s performance improves as the fitness value decreases, since the goal is to minimize the objective function. The BKAPI (magenta line), such as F1, F5, F6, F9, F10, F20, F23, and F29 with dimensions of 10 (Figure 4) and F1, F5, F6, F9, F21, and F30 with dimensions of 100 (dark wine red line) (Figure 5), shows the fastest and most effective convergence, rapidly reaching the minimum fitness value across most functions. Algorithms like the BKA and PSO exhibit slower convergence and higher final fitness values, indicating suboptimal performance. As for other curves, the performance of the BKAPI in certain functions is not the best, which is normal. Overall, the BKAPI consistently demonstrates superior convergence speed and optimization accuracy across all tested functions, making it the most robust and effective among the compared methods. Other algorithms, such as the BKA and AO, show moderate performance, while methods like the GA, PSO, and BWO generally underperform.

**Figure 6:**
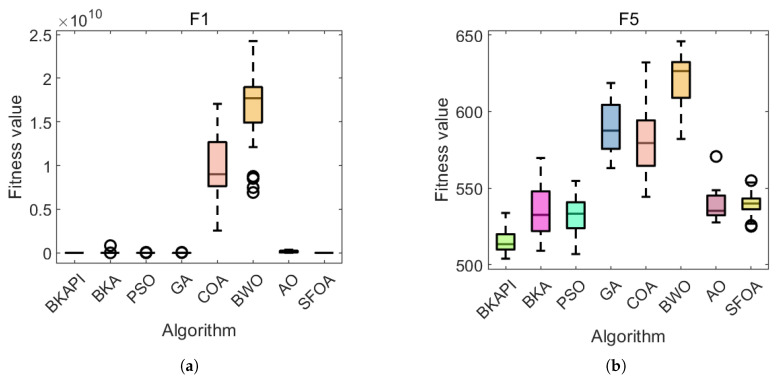

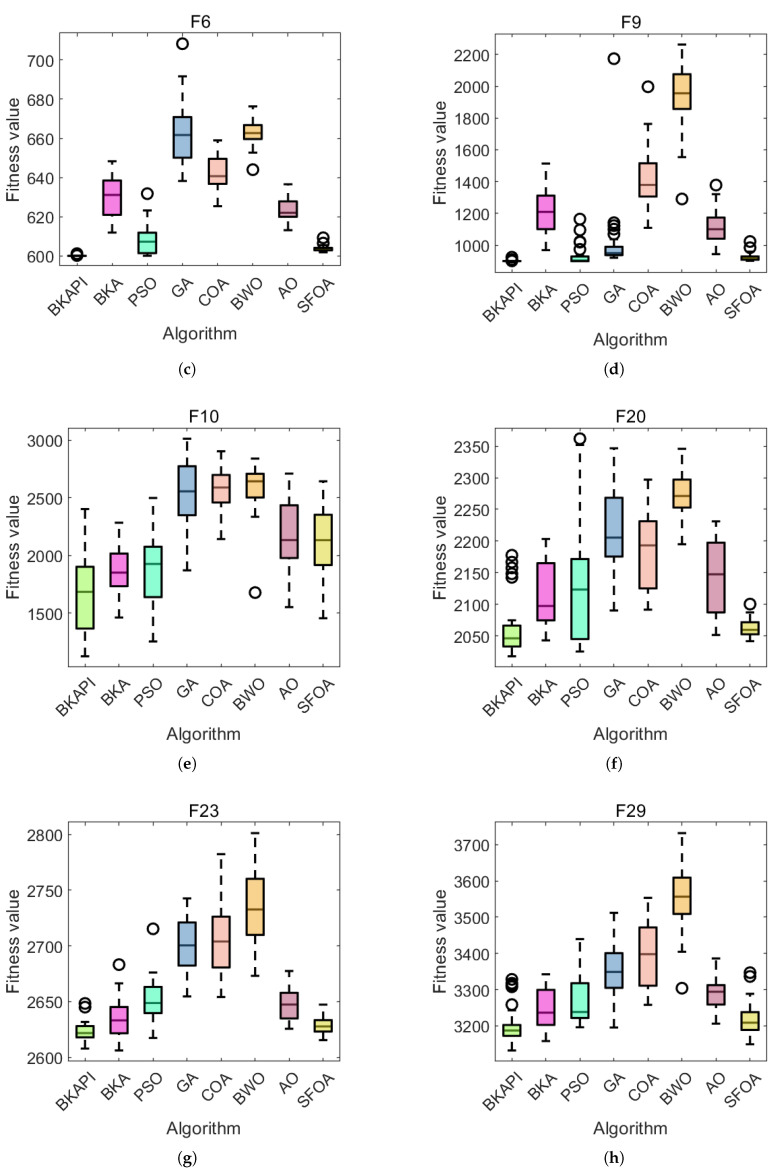
Parts of the boxplot comparing the proposed BKAPI and competitor algorithms on selected functions of the CEC 2017 benchmark with dimensions of 10 (Circles represent outliers). (**a**) f1, (**b**) f5, (**c**) f6, (**d**) f9, (**e**) f10, (**f**) f20, (**g**) f23, (**h**) f29.

**Figure 7:**
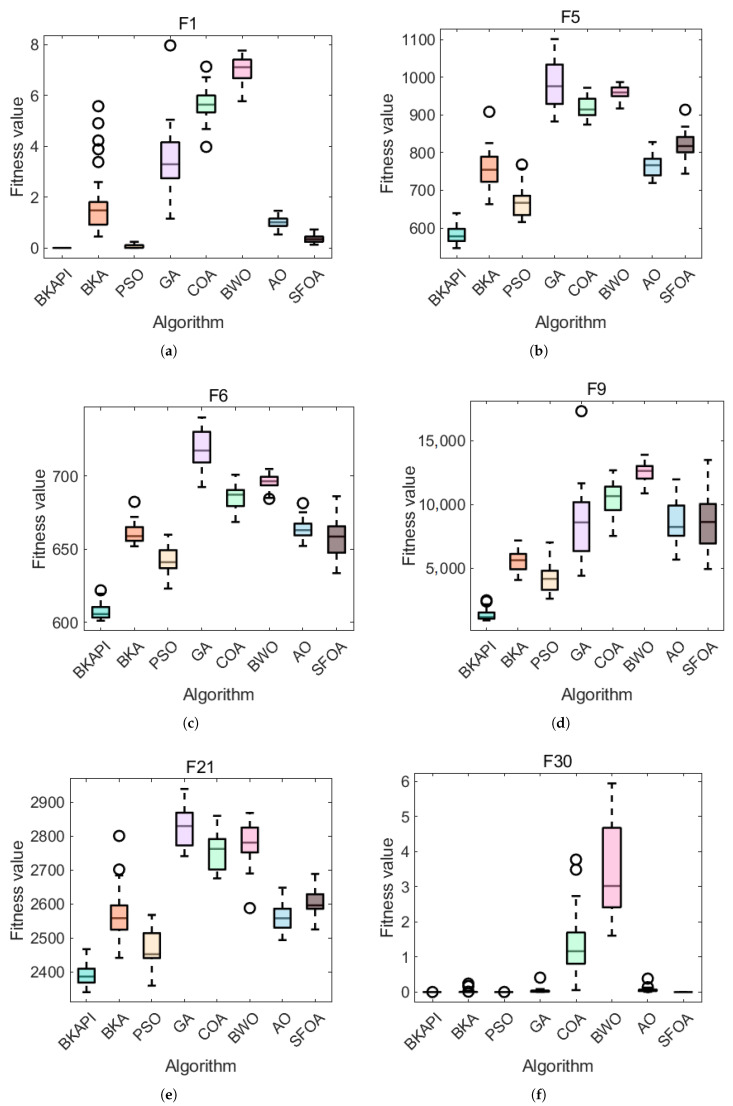
Parts of the boxplot comparing the proposed BKAPI and competitor algorithms on selected functions of the CEC 2017 benchmark with dimensions of 100 C. (**a**) f1, (**b**) f5, (**c**) f6, (**d**) f9, (**e**) f21, (**f**) f30.

Parts of the ANOVA test results with dimensions of 10 are presented in Figure 6, while those with dimensions of 100 are presented in Figure 7. The standard deviation acts as a clear indicator of each algorithm’s stability in solving optimization problems; a smaller standard deviation reflects stronger overall optimization ability and stability. Consequently, the algorithms are ranked based on their standard deviations. The BKAPI integrates a hybrid algorithm strategy and a mutation strategy, which enhance its performance, resulting in improved convergence rates and calculation precision. For functions F1–F3, the BKAPI exhibits the smallest standard deviation among all algorithms, ranking it first. This indicates its strong stability, superior optimization ability, and practicality in solving unimodal functions. For functions F4–F10, the BKAPI employs additional strategies to expand the population space and avoid local optima, enhancing both its global and local search abilities. Its standard deviation remains smaller than those of other algorithms, further demonstrating its stability. Similarly, for functions F11–F20, BKAPI demonstrates strong overall search capabilities, enabling it to reliably identify precise solutions within the search space. Its performance, characterized by a low standard deviation, underscores its superiority and stability. For F21 and F23, the BKAPI and BKA are clearly dominant, with minimal variation and consistently low fitness values. For F24 to F26, the trend continues, with the BKAPI maintaining its edge. The SFOA occasionally shows competitive results but lacks consistency. For functions F27–F30, the BKAPI maintains consistent stability with relatively small standard deviations compared to the other algorithms. This stability highlights its effectiveness in tackling complex function problems. Metrics such as the mean value, standard deviation, and *p*-value are used as evaluation indicators to further verify its robustness. The robustness of BKAPI is evident in several aspects: it balances exploration and exploitation to achieve faster convergence and higher precision, maintains a relatively small standard deviation indicative of strong optimization ability, and ensures that even in cases of higher deviation, catastrophic or combinatorial failures are avoided.

**Figure 8:**
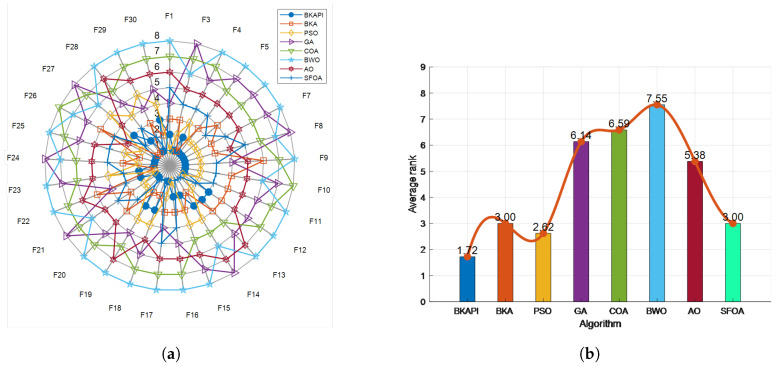
Sorting diagram of BKAPI’s performance compared to other algorithms on the CEC 2017 test set with dimensions of 10. (**a**) The radar chart. (**b**) The comparison chart of algorithm performance based on average rank.

**Figure 9:**
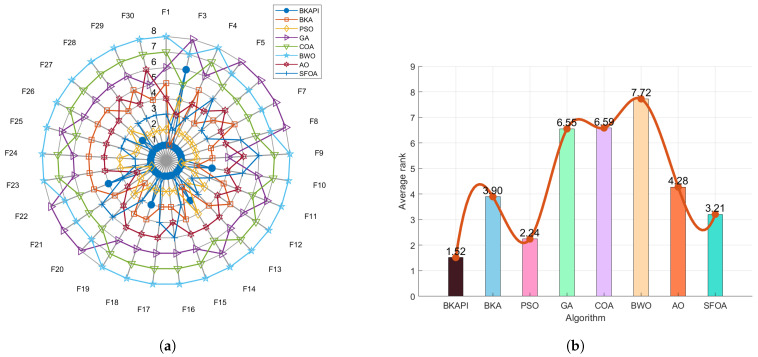
Sorting diagram of BKAPI’s performance compared to other algorithms on the CEC 2017 test set with dimensions of 100. (**a**) The radar chart. (**b**) The comparison chart of algorithm performance based on average rank.

Figure 8a shows a radar chart illustrating the comparison of other algorithms across 29 test functions in with dimensions of 10, while Figure 9a shows the same with dimensions of 100. Each axis corresponds to a specific test function, and the larger the value along an axis, the worse the algorithm’s performance. The smaller overall shape in the radar chart indicates better performance. Different colors and symbols represent the algorithms, with the BKAPI (blue dots) exhibiting the smallest enclosed area, signifying superior performance for most test functions.

The comparison chart of algorithm performance based on the average ranks of the algorithms with dimensions of 10, where a smaller ranking value reflects better performance, is shown in Figure 8b. The x-axis (algorithm) lists different optimization algorithms, namely the BKAPI, BKA, PSO, GA, COA, BWO, AO, and SFOA, while the y-axis (average rank) represents the average ranking of each algorithm, and bars show the average rank of each algorithm, with different colors representing different algorithms. A trend line that connects the ranking values provides a visual representation of ranking changes across algorithms. Among all algorithms, the BKAPI achieves the best results, consistently ranking the lowest, with 1.72 with dimensions of 10 and 1.52 with dimensions of 100 (Figure 9b). The BKAPI has the best performance, while the SFOA (Starfish Optimization Algorithm) has a mid-range performance (its rank is 3.21 with dimensions of 100), indicating its strong optimization capabilities. PSO performs competitively but falls slightly behind the BKA and SFOA (its rank is 2.24 with dimensions of 100). In contrast, the GA, COA, AO, and BWO demonstrate relatively poor performance, with higher average rankings across the test functions. While these algorithms may excel in certain specific functions, their overall average rankings are not as favorable as those of the BKAPI, BKA, and SFOA.

Overall, BKAPI showcases significant advantages in optimization, reflecting strong and stable performance across multiple test functions. These observations are consistent with the results presented in Table 1, Table 2 and Table 3 and Figure 4, Figure 5, Figure 6, Figure 7 and Figure 8 for dimensions of 10 and Figure 5, Figure 6, Figure 7, Figure 8 and Figure 9 for dimensions of 100, further confirming the BKAPI’s dominance and stability in function optimization tasks in high-dimensional spaces.

### 4.3. Results and Analysis of the Test Functions for CEC 2022

The CEC 2022 test function has 12 single-target test functions (like CEC 2017): a single-peak function (F1), basic functions (F2–F5), mixed functions (F6–F8), and combination functions (F9–F12) [54]. Table 4 shows the standard deviation and average fitness value results of the CEC 2022 test set.

The BKAPI achieves the lowest mean values and standard deviation values across most functions, which indicates its superior efficiency and robustness in optimization. It excels particularly in F1 (avg: 3.00 $\times \;10^{2}$, std: 1.07 $\times \;10^{-10}$) and F5 (avg: 9.01 $\times \;10^{2}$, std: 1.48 $\times \;10^{0}$). The SFOA emerges as a strong competitor, especially in F8, F10, F11, and F12, demonstrating excellent consistency (e.g., F12: std: 1.65 $\times \;10^{0}$). The BKA and PSO show competitive but less consistent results, trailing the BKAPI in most cases. Algorithms like the GA, COA, and BWO generally underperform, with higher avg and std values, highlighting their instability in handling complex functions.

We analyze Table 5 for values greater than 0.05 (5 $\times \;10^{-2}$) by identifying and comparing cases where the *p*-value exceeds this threshold. From the data, it is observed that the GA, COA, BWO, and AO frequently achieve the smallest *p*-values, at 3.02 $\times \;10^{-11}$, indicating that the BKAPI demonstrates statistically significant differences for the corresponding test functions.

**Figure 10:**
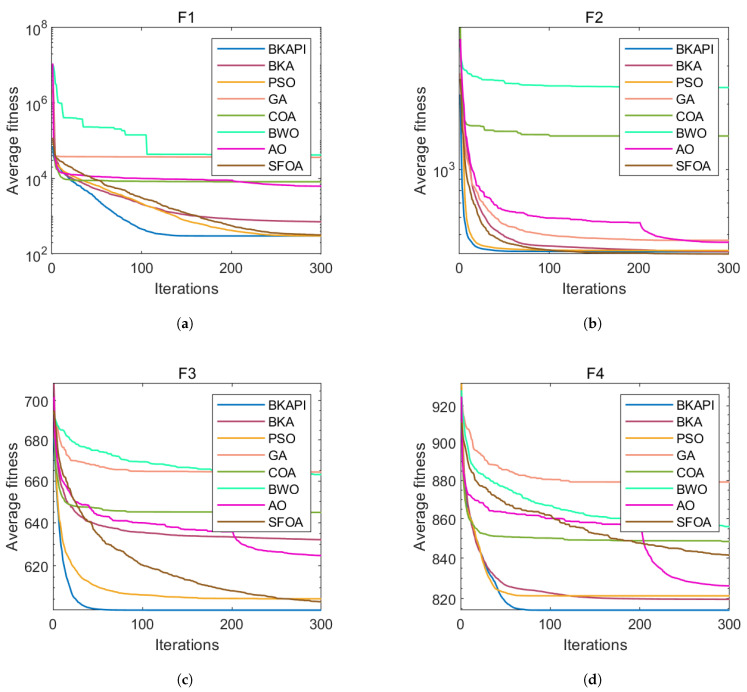

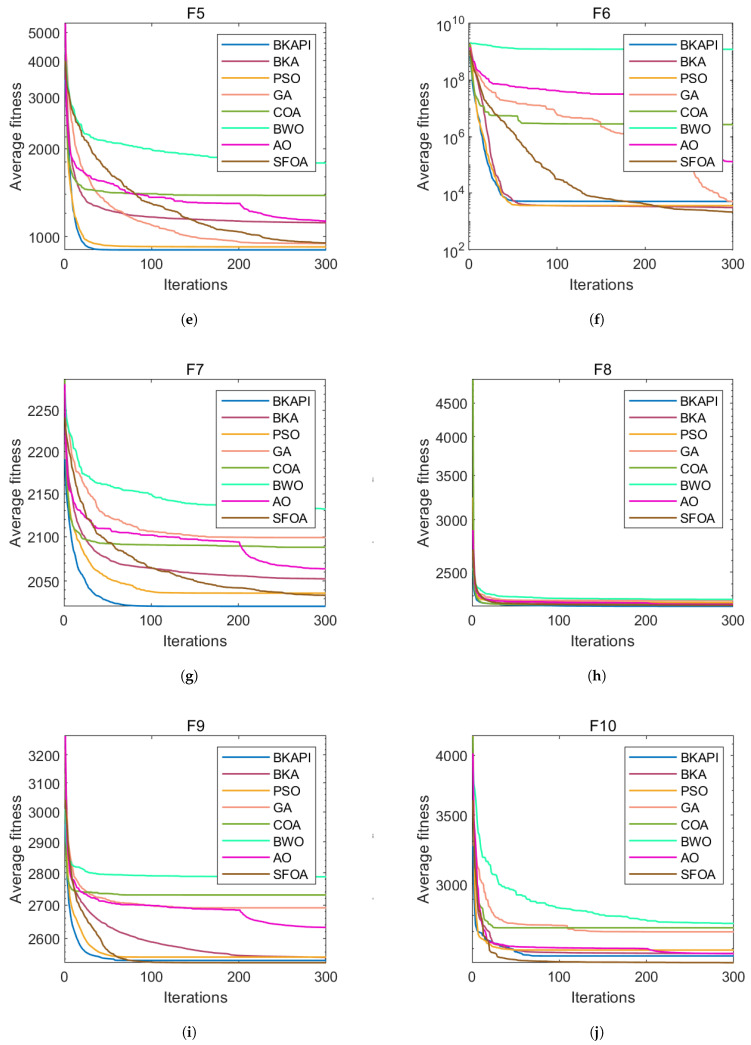

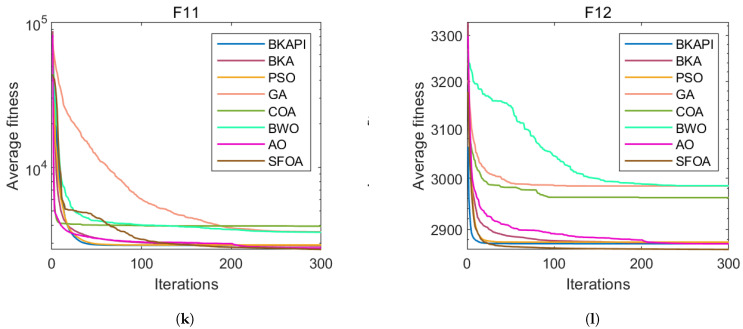
Convergence analysis of the BKAPI and competing algorithms on selected functions of the CEC 2022 benchmark with dimensions of 10. (**a**) f1, (**b**) f2 (**c**) f3, (**d**) f4, (**e**) f5, (**f**) f6, (**g**) f7, (**h**) f8, (**i**) f9, (**j**) f10, (**k**) f11, (**l**) f12.

**Figure 11:**
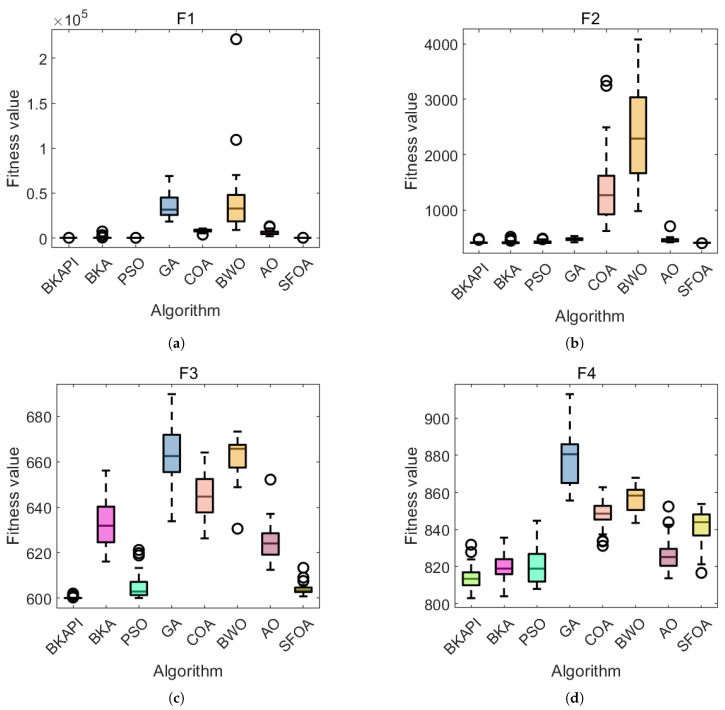

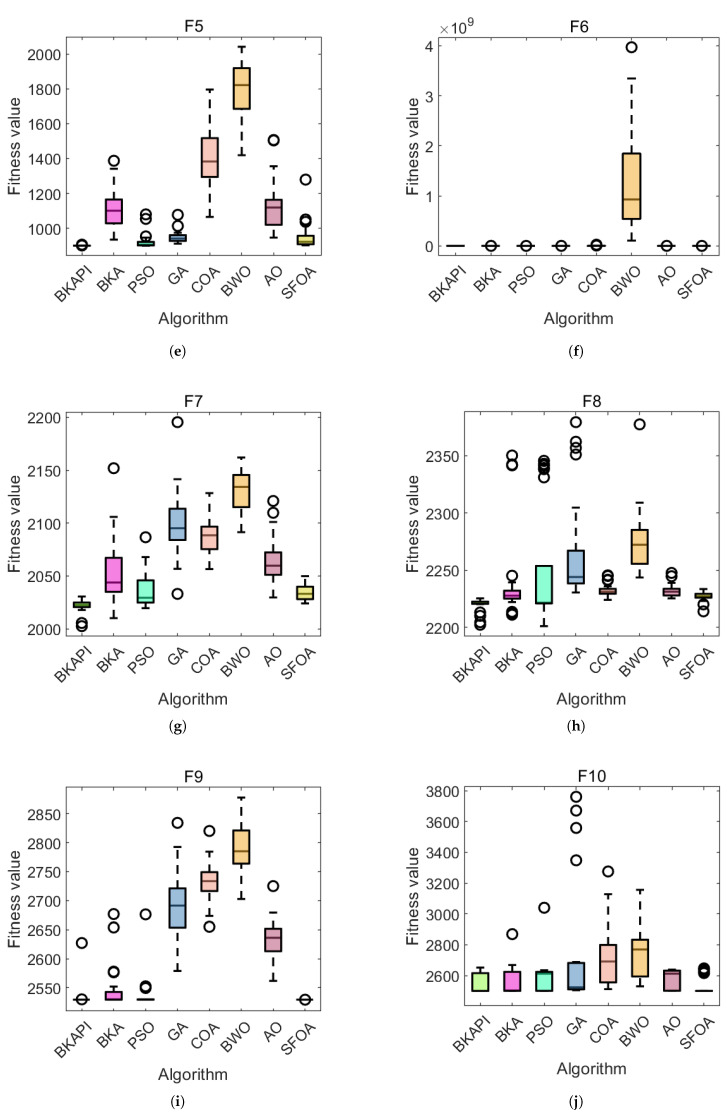

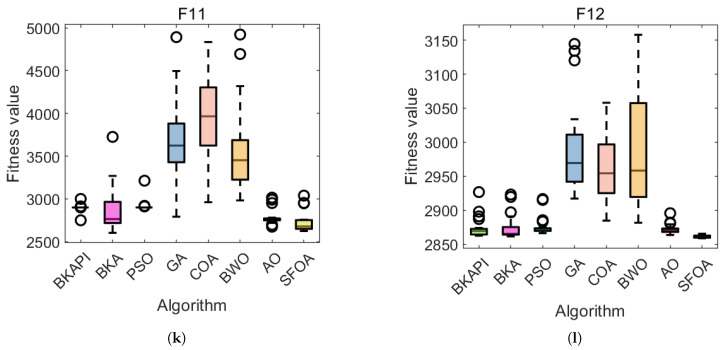
Boxplot comparing the performance of the BKAPI and competitor algorithms on selected functions of the CEC 2022 benchmark with dimensions of 10 (Circles represent outliers). (**a**) f1, (**b**) f2 (**c**) f3, (**d**) f4, (**e**) f5, (**f**) f6, (**g**) f7, (**h**) f8, (**i**) f9, (**j**) f10, (**k**) f11, (**l**) f12.

The results of the convergence curve and ANOVA test on partial functions of CEC 2022 for the algorithms are presented in Figure 10 and Figure 11. The BKAPI exhibits the fastest convergence speed and optimal fitness value among all test functions, making it the most stable and effective algorithm. The BKA also exhibits good performance but is slightly inferior to the BKAPI in some functions. PSO and the GA perform well on most functions, but not as well as the BKAPI and BKA. The COA, BWO, AO, and SFOA perform poorly on most functions. These analyses indicate that the BKAPI is the most reliable and effective algorithm suitable for these test functions.

**Figure 12:**
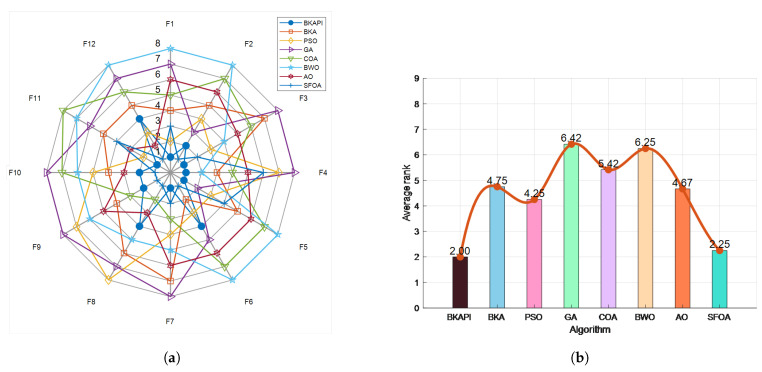
Sorting diagram of the BKAPI’s performance compared to other algorithms on the CEC 2022 test set with dimensions of 10. (**a**) The radar chart. (**b**) The comparison chart of algorithm performance based on average rank.

The radar chart illustrates the performance comparison of various optimization algorithms across 12 test functions, as shown in Figure 12a. Each algorithm is represented by distinct colors and symbols, with the BKAPI (denoted by blue dots) showing the smallest enclosed area, which signifies its superior performance across most of the test functions. Figure 12b presents the average ranking of the algorithms, where a lower ranking number reflects better overall performance. Among all the algorithms evaluated, the BKAPI stands out with the best results, maintaining a consistently low rank of 2.00. The BKA and SFOA also perform admirably, achieving relatively low average rankings that highlight their robust optimization capabilities. PSO demonstrates competitive performance but ranks slightly below the BKA and SFOA. In contrast, the GA, COA, AO, and BWO exhibit relatively weaker performance, as evidenced by their higher average rankings. Although these algorithms might excel on specific functions, their overall performance does not match the consistency and effectiveness of the BKAPI, BKA, and SFOA.

In summary, the BKAPI exhibits significant advantages in optimization tasks, showcasing its strong and stable performance across multiple test functions. The consistent top rankings of the BKAPI are further supported by the data in Table 4 and Table 5, as well as Figure 10 and Figure 11. The BKA and SFOA also prove to be versatile and reliable optimization algorithms, suitable for diverse application scenarios. This analysis underscores the BKAPI’s dominance and stability in optimizing a wide range of functions. Building on these promising results, the next step is to validate the BKAPI’s effectiveness in real-world engineering problems, where complex constraints and practical challenges further test its robustness and applicability.

## 5. Engineering Optimization Application

The BKAPI is applied to optimization problems for classic engineering verification (welded beam project and Himmelblau function) and actual engineering applications (visible light positioning (VLP) system) to further demonstrate the effectiveness of improvement strategies and the engineering applicability of the BKAPI. This section will present the problem formulation, the application of the BKAPI, and the results obtained, emphasizing its performance in tackling these challenging engineering optimization tasks.

### 5.1. Welded Beam Project

**Figure 13:**
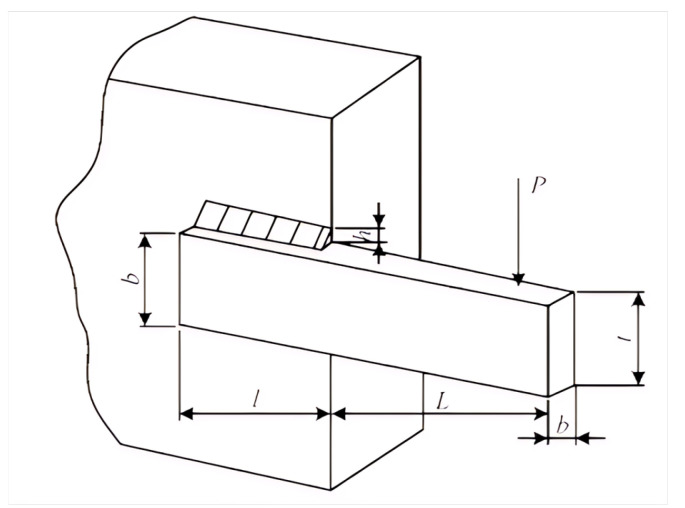
Welded beam design project.

The objective of the welded beam design problem is to minimize the cost function while satisfying a set of seven inequality constraints (for details on the relevant introduction and calculation formulas, see [55]). The objective of the welded beam design problem is to minimize the cost function f(x) while adhering to a set of seven inequality constraints. These constraints include limitations on the shear stress (τ), beam bending stress (σ), buckling load of the bar ($P_{C}$), end deflection of the beam (δ), and others. As illustrated in Figure 13, the design involves four variables: the height of the weld ($h(x_{1})$), the length of the weld ($l(x_{2})$), the thickness of the beam ($t(x_{3})$), and the width of the beam ($b(x_{4})$) [55].

Mathematically, the optimization model can be formulated as follows:

Consider variable $x=\left[x_{1},x_{2},x_{3},x_{4}\right]=\left[h,l,t,b\right]$

Minimize

(10) $$\;\min f\left(x\right)=1.1047x_{1}^{2}x_{2}+0.04811x_{3}x_{4}\left(14.0+x_{2}\right)|\;$$

subject to

(11) $$\;g_{1}\left(x\right)=\tau \left(x\right)-\tau_{\max}\leq 0\;$$

(12) $$\;g_{2}\left(x\right)=\sigma \left(x\right)-\sigma_{\max}\leq 0\;$$

(13) $$\;g_{3}\left(x\right)=x_{1}-x_{4}\leq 0\;$$

(14) $$\;g_{4}\left(x\right)=0.104x_{1}^{2}+0.04811x_{3}\;$$

(15) $$\;x_{4}\left(14.0+x_{2}\right)-5.0\leq 0\;$$

(16) $$\;g_{5}\left(x\right)=0.125-x_{1}\leq 0\;$$

(17) $$\;g_{6}\left(x\right)=\delta \left(x\right)-\delta_{\max}\leq 0\;$$

(18) $$\;g_{7}\left(x\right)=P-P_{c}\left(x\right)\leq 0$$

Boundary constraints and related parameters:

(19) $$\;\tau \left(x\right)=\sqrt{{\left(\tau^{\prime }\right)}^{2}+2\tau^{\prime }\tau^{\prime \prime }\frac{x_{2}}{2R}+{\left(\tau^{\prime \prime }\right)}^{2}}\;$$

(20) $$\;\tau^{\prime }=\frac{P}{\sqrt{2}x_{1}x_{2}}\;$$

(21) $$\;\tau^{\prime \prime }=\frac{MR}{J}\;$$

(22) $$\;M=P\left(L+\frac{x_{2}}{2}\right)\;$$

(23) $$\;R=\sqrt{\frac{x_{2}^{2}}{4}+{\left(\frac{x_{1}+x_{3}}{2}\right)}^{2}}\;$$

(24) $$\;J=2\left[\sqrt{2}x_{1}x_{2}\left\{\frac{x_{2}^{2}}{12}+{\left(\frac{x^{1}+x^{3}}{2}\right)}^{2}\right\}\right]\;$$

(25) $$\;\sigma \left(x\right)=\frac{6PL}{x_{3}^{2}x_{4}},\delta \left(x\right)=\frac{4PL^{3}}{Ex_{3}^{3}x_{4}}\;$$

(26) $$\;P_{c}\left(x\right)=\frac{4.013E\sqrt{\frac{x_{3}^{2}x_{4}^{6}}{36}}}{L^{2}}\left(1-\frac{x_{3}}{2L}\sqrt{\frac{E}{4G}}\right)\;$$

(27) $$\;P=6000\;\mathrm{lb},L=14\;\mathrm{in},E=30\times 10^{6}\;\mathrm{psi},G=12\times 10^{6}\;\mathrm{psi},\;$$

(28) $$\;\tau_{\max}=13,600\;\mathrm{psi},\sigma_{\max}=30,000\;\mathrm{psi},\delta_{\max}=0.25\;\mathrm{in}\;$$

(29) $$\;0.1\leq x_{1}\leq 2.0,0.1\leq x_{2}\leq 10.0,\;$$

(30) $$\;0.1\leq x_{3}\leq 10.0,0.1\leq x_{4}\leq 2.0\;$$

**Figure 14:**
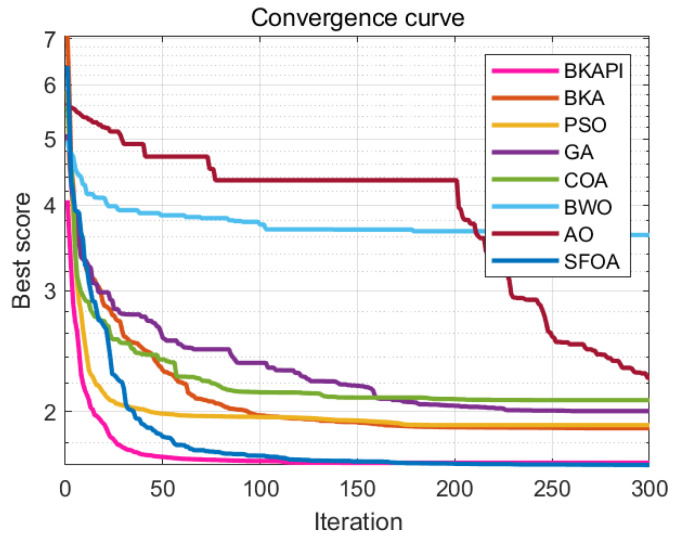
The average convergence curve of the welded beam project.

The BKAPI was applied to solve the welding beam project problem, with the following parameter settings: a population size of 30, a maximum of 300 iterations, a movement probability of 0.9, and an exchange probability of 0.9. The algorithm was executed independently to obtain the optimal value, average value, and function evaluation counts for the objective function. The convergence curve and index statistics are shown in Figure 14 and Table 6, respectively. All algorithms demonstrate good convergence speed, and the BKAPI, PSO, and SFOA show good global optimization capabilities in Figure 14. The standard deviation of the BKAPI is 0.05, which is much better than the corresponding value of 0.65 for the BKA. The data in Table 6 reveal that the BKAPI achieved the best results in both the optimal value and average value across 30 independent runs while also requiring the fewest function evaluations. The results in Table 7 show that the BKAPI obtains the best optimal value, at 1.6702, indicating that BKAPI can provide the best solutions for the welding beam project problem. The BKAPI is the best choice if accuracy and stability are the top priorities, despite its slower computational time (2.95 s). In conclusion, the BKAPI and SFOA stand out as the most effective algorithms, balancing optimization accuracy, consistency, and computational efficiency.

### 5.2. Himmelblau Function

D. M. Himmelblau cited this problem to simulate process design challenges in his work [56]. It has since become a widely used benchmark for evaluating nonlinear constrained optimization algorithms. This problem involves five variables and six nonlinear constraints, with its detailed description provided in [57].

Minimize

(31) $$\;f\left(\bar{x}\right)=5.3578547x_{3}^{2}+0.8356891x_{1}x_{5}+37.293239x_{1}-40792.141\;$$

subject to

(32) $$\;g_{1}\left(\bar{x}\right)=-G1\leq 0,\;\;g_{2}\left(\bar{x}\right)=G1-92\leq 0,\;$$

(33) $$\;g_{3}\left(\bar{x}\right)=90-G2\leq 0\;$$

(34) $$\;g_{4}\left(\bar{x}\right)=G2-110\leq 0,\;$$

(35) $$\;g_{5}\left(\bar{x}\right)=20-G3\leq 0,\;$$

(36) $$\;g_{6}\left(\bar{x}\right)=G3-25\leq 0,\;$$

where

(37) $$\;G1=85.334407+0.0056858x_{2}x_{5}+0.0006262x_{1}x_{4}-0.0022053x_{3}x_{5},\;$$

(38) $$\;G2=80.51249+0.0071317x_{2}x_{5}+0.0029955x_{1}x_{2}+0.0021813x_{3}^{2},\;$$

(39) $$\;G3=9.300961+0.0047026x_{3}x_{5}+0.00125447x_{1}x_{3}+0.0019085x_{3}x_{4}.\;$$

within the bounds of

(40) $$\;78\leq x_{1}\leq 102,\;$$

(41) $$\;33\leq x_{2}\leq 45,\;$$

(42) $$\;27\leq x_{3}\leq 45,\;$$

(43) $$\;27\leq x_{4}\leq 45,\;$$

(44) $$\;27\leq x_{5}\leq 45$$

**Figure 15:**
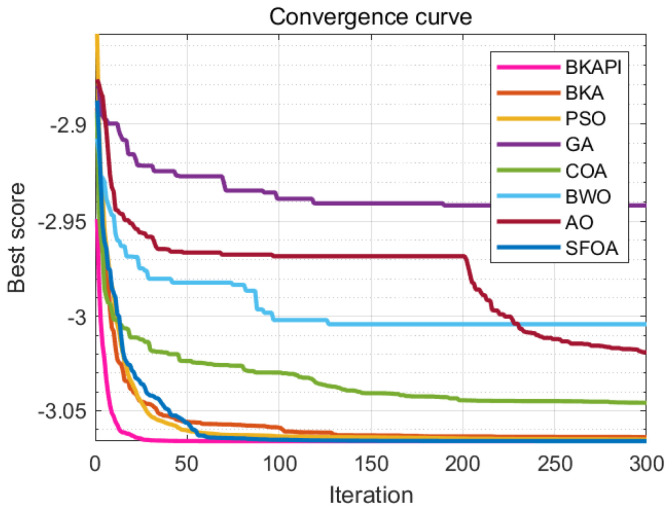
The average convergence curve of the Himmelblau function optimization.

The BKAPI was applied to solve this problem by using the following parameter settings: a population of 30 and 300 iterations and 30 independent runs to obtain the optimal value, average value, and function evaluation counts for the objective function [58]. The outcomes were evaluated against seven alternative algorithms, with the convergence trends illustrated in Figure 15 and the corresponding performance metrics detailed in Table 8 and Table 9. All algorithms exhibit good convergence speeds, with the BKAPI, BKA, and SFOA demonstrating strong global optimization capabilities in Figure 15. The standard deviation is −30,186,151 and the average value is −30,617 for the BKAPI, respectively, demonstrating a significant advantage over the other algorithms. The computation time of the BKAPI (3.68 s) was on the slower side, but its superior performance may justify the extra computational time. These data indicate that the BKAPI achieved the best performance in terms of both the optimal value and the average value over 30 independent runs, while also requiring the lowest number of function evaluations in Table 9.

While the proposed BKAPI demonstrates significant improvements in global exploration, local search capabilities, and solution quality, it is important to acknowledge and discuss its limitations. One key limitation is its computational complexity, which arises from the integration of multiple mechanisms, including PSO’s velocity-update rule and the Random-Elite Difference Mutation strategy. These components, while enhancing performance, increase the computational overhead compared to the basic BKA algorithm. Additionally, the BKAPI’s performance is sensitive to hyperparameter settings, such as the swarm size in PSO and mutation rates. The improper tuning of these parameters can result in suboptimal performance or increased computational costs.

To address these limitations, a runtime analysis was conducted to compare the computational efficiency of the BKAPI with other state-of-the-art algorithms, including the basic BKA, PSO, GA, and SFOA. The analysis was performed on the CEC 2017 and CEC 2022 benchmark functions, which include a variety of high-dimensional and complex optimization problems. The results indicate that BKAPI, while slightly more computationally expensive than the basic BKA, achieves faster convergence rates and higher solution accuracy. This trade-off between computational cost and solution quality is justified, particularly for complex and multimodal problems where traditional algorithms often struggle.

### 5.3. Visible Light Positioning (VLP) System

Visible light communication (VLC) technology has advanced rapidly in recent years, leveraging light-emitting diodes (LEDs) for data transmission. VLC offers benefits, making it a promising solution for indoor positioning. Visible light positioning (VLP) systems improve LED utilization and enable high-precision positioning. These systems are divided into two categories based on the receiver type: image sensor-based systems, which use imaging and geometric relationships for positioning, and photodiode (PD)-based systems, which rely on methods like TOA, TDOA, AOA, and RSS to calculate distances or angles and estimate locations through triangulation [59,60,61].

This study used MATLAB as an experimental platform to design an experimental framework for a visible light indoor positioning algorithm system to evaluate and compare the performance of different single-point estimation algorithms based on received signal intensity (RSS). The experimental space was set to a three-dimensional area of 5 m × 5 m × 2 m, and the sampling points were generated by arranging 0.5 m on the x- and y-axes and a fixed z-axis at 2 m. This sampling method guarantees a uniform distribution of test points in space, enabling a comprehensive assessment of the performance in various locations [62]. To guarantee the dependability of the experimental outcomes, the two algorithms were run separately at each sampling point, and their estimated positions and positioning errors were recorded.

During the data acquisition process, a single-point estimation function was used to perform single-point estimation. This took the sample point coordinates as the input and returned the estimated position and positioning error. By looping through all sample points, the performance data of the two algorithms on each point were collected. These data included estimated position coordinates and corresponding positioning errors, laying the foundations for subsequent performance evaluation and visual analysis.

The location results of the BKA and BKAPI on all sampling points were obtained. First, we calculated the average positioning error for both algorithms through the collection and processing of experimental data. The results indicate that the average error of the BKAPI is 2.48148 cm, which is significantly better than the 8.10665 cm error for the BKA algorithm. This result shows that the position improvement mechanism introduced in the BKAPI effectively improves the positioning accuracy.

**Figure 16:**
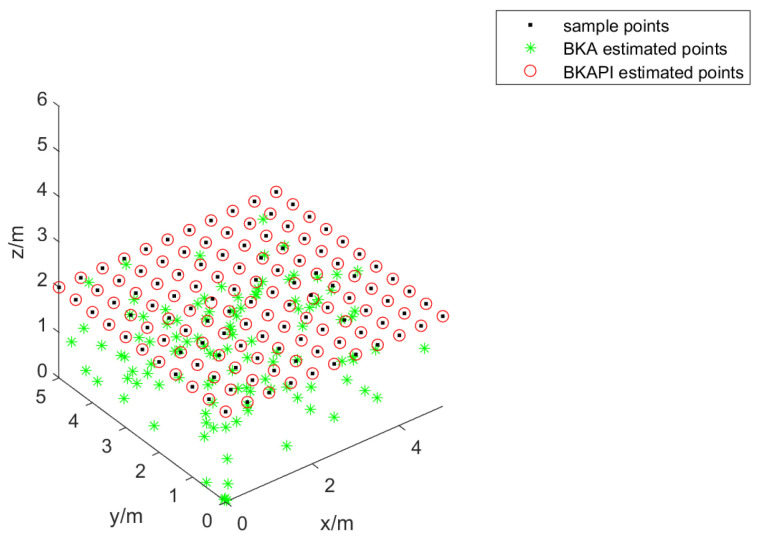
Sample positions and their estimated positions for the BKA and BKAPI.

To provide a more intuitive demonstration of the comparison, a three-dimensional point cloud visualization was created. Figure 16 shows the distribution of sampling points, BKA estimation points, and BKAPI estimation points in three-dimensional space. As shown in the figures, the BKAPI estimation points (green asterisk) are closer to the actual sampling points (black dot) compared to the BKA estimation points (red circle), further confirming the superiority of the BKAPI.

**Figure 17:**
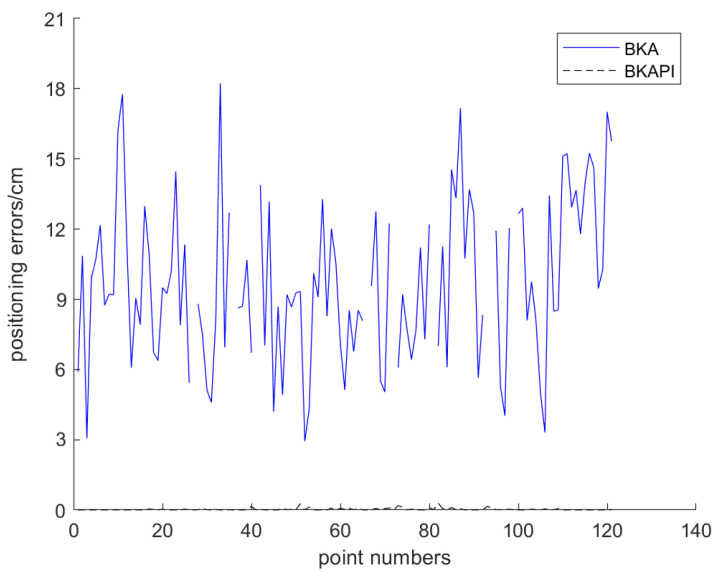
The positioning error curves.

In terms of error analysis, the positioning error curves of the two algorithms at all sampling points were drawn (Figure 17). The error curve (blue solid line) of the BKAPI is located below that of the BKA algorithm (black dashed line), indicating that the BKAPI exhibits better positioning accuracy at most sampling points. The errors of both algorithms in certain areas are relatively large, which may be related to signal propagation characteristics or environmental interference and thus deserve further research.

**Figure 18:**
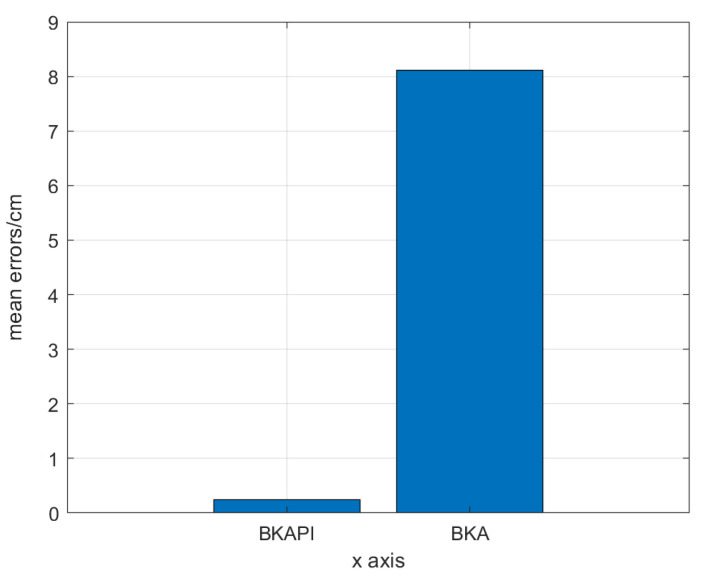
The bar chart of the positioning mean error.

Finally, Figure 18 presents a bar chart comparing the average errors of the two algorithms; the error for the BKAPI is significantly lower than that for the BKA, and this result aligns with the previous analysis. Through this multi-dimensional performance evaluation and visual analysis, we not only verify the superiority of the BKAPI but also provide an important reference for subsequent algorithm improvements.

The experimental findings reveal that the BKAPI significantly outperforms the BKA in positioning accuracy, exhibiting a notably lower average positioning error. Through three-dimensional point cloud visualization and error analysis, the superiority and stability of BKAPI across different spatial locations are further confirmed.

In summary, while the BKAPI introduces additional computational complexity and requires careful hyperparameter tuning, its enhanced performance and versatility make it a promising approach for solving challenging optimization problems. Future work could focus on developing adaptive mechanisms to reduce hyperparameter sensitivity and further optimize computational efficiency.

## 6. Conclusions and Prospects

This article introduces an enhanced Black-Winged Kite Algorithm (BKA) integrated with Particle Swarm Optimization (PSO) that is designed to address functional optimization and engineering challenges. By combining the BKA’s search mechanisms with PSO’s global search capabilities, local development potential, and Random-Elite Difference Mutation strategy, the hybrid BKAPI achieves greater population diversity and prevents premature convergence. The BKAPI demonstrates reduced dependency on initial populations, adaptability to various optimization problem types, and versatility in solving continuous, discrete, and combinatorial optimization problems, thereby improving its robustness, adaptability, and broad applicability.

In benchmark testing for CEC 2017 and CEC 2022, the BKAPI demonstrates superior performance with regard to both the standard deviation and average results, highlighting its robust stability and global optimization capabilities. Convergence curve analysis shows that BKAPI significantly enhances global optimization ability and robustness, outperforming existing methods in engineering optimization problems. The experimental results confirm that the BKAPI improves both convergence speed and accuracy, making it highly suitable for practical engineering applications. These advancements position the hybrid algorithm as an effective solution for complex optimization tasks, such as indoor positioning and outdoor path planning, offering improved performance, greater solution efficiency, and enhanced robustness.

Future research directions include further optimization of the algorithm through additional channels, such as integrating it with deep learning models or testing it on large-scale industrial datasets. Building on our prior research in indoor positioning, the BKAPI can be applied to engineering applications like indoor positioning and robotic path planning. Specific recommendations for future work include the following:

1. Algorithm Enhancement: The BKAPI faces challenges related to computational cost, scalability, parameter sensitivity, and premature convergence. Future research should focus on adaptive parameter tuning, hybridization with machine learning, parallel computing, and theoretical analysis to address these limitations and enhance its performance.
2. Large-Scale Testing: The algorithm’s performance should be validated on large-scale industrial datasets to assess its scalability and efficiency in real-world scenarios.
3. Extension to Constrained Optimization: The BKAPI should be extended to handle constrained optimization problems, which are ubiquitous in real-world applications. This could involve incorporating constraint-handling techniques such as penalty functions, feasibility rules, or multi-archive strategies, as discussed in recent works [63,64].

By pursuing these directions, the BKAPI can be further refined and applied to a broader range of complex optimization challenges, solidifying its practical relevance and impact. 

## Figures and Tables

**Table 1:** CEC 2017 test set standard deviation value results with dimensions of 10.

Func.	BKAPI	BKA	PSO	GA	COA	BWO	AO	SFOA
F1	5.0464 × 10^3^	1.5145 × 10^8^	1.2790 × 10^7^	1.6664 × 10^7^	3.8116 × 10^9^	4.4097 × 10^9^	1.1448 × 10^8^	1.8762 × 10^5^
F3	3.7433 × 10^−11^	1.6003 × 10^3^	3.9216 × 10^1^	2.1379 × 10^4^	2.8050 × 10^3^	2.9759 × 10^3^	1.3958 × 10^3^	1.1623 × 10^1^
F4	2.8080 × 10^1^	7.6158 × 10^1^	2.2099 × 10^1^	5.5817 × 10^1^	5.5150 × 10^2^	6.1985 × 10^2^	3.6989 × 10^1^	9.4219
F5	7.3213	1.6916 × 10^1^	1.3267 × 10^1^	1.6580 × 10^1^	2.3751 × 10^1^	1.6905 × 10^1^	9.1983	7.1405
F6	2.1839 × 10^−1^	1.0964 × 10^1^	7.7139	1.5626 × 10^1^	8.7581	6.5523	6.5114	1.4954
F7	6.1033	2.1250 × 10^1^	1.0046 × 10^1^	3.8370 × 10^1^	2.4738 × 10^1^	1.4120 × 10^1^	1.2983 × 10^1^	1.4490 × 10^1^
F8	7.3601	9.2051	1.0148 × 10^1^	1.2611 × 10^1^	8.6554	6.7476	8.9182	8.5151
F9	4.7548	1.2694 × 10^2^	6.3308 × 10^1^	2.2671 × 10^2^	1.8608 × 10^2^	2.1063 × 10^2^	1.0356 × 10^2^	2.6600 × 10^1^
F10	3.2500 × 10^2^	1.8461 × 10^2^	3.1883 × 10^2^	2.8035 × 10^2^	1.7675 × 10^2^	2.1546 × 10^2^	3.3261 × 10^2^	3.3115 × 10^2^
F11	4.8805 × 10^1^	4.2515 × 10^1^	3.7330 × 10^1^	8.3714 × 10^3^	1.2837 × 10^3^	5.9459 × 10^3^	4.0757 × 10^2^	6.3904
F12	1.7226 × 10^6^	7.0995 × 10^5^	1.4927 × 10^6^	5.1580 × 10^6^	2.0073 × 10^8^	4.8231 × 10^8^	4.2216 × 10^6^	1.2198 × 10^4^
F13	8.9421 × 10^3^	1.3062 × 10^3^	7.4830 × 10^3^	1.0243 × 10^4^	1.0985 × 10^4^	3.5018 × 10^7^	1.2377 × 10^4^	2.3808 × 10^2^
F14	1.3225 × 10^2^	4.2050 × 10^1^	4.3146 × 10^3^	7.8841 × 10^3^	2.9085 × 10^1^	3.4428 × 10^2^	1.6960 × 10^3^	4.9984
F15	1.8812 × 10^3^	8.3161 × 10^1^	5.8859 × 10^3^	8.8665 × 10^3^	3.6689 × 10^3^	2.3477 × 10^3^	5.6589 × 10^3^	2.6217 × 10^1^
F16	1.1473 × 10^2^	9.8940 × 10^1^	1.4577 × 10^2^	1.3478 × 10^2^	1.4880 × 10^2^	1.1463 × 10^2^	1.3996 × 10^2^	5.8275 × 10^1^
F17	5.4768 × 10^1^	2.1348 × 10^1^	6.0177 × 10^1^	3.5123 × 10^1^	3.5999 × 10^1^	3.4504 × 10^1^	2.0671 × 10^1^	1.5171 × 10^1^
F18	1.6189 × 10^4^	4.5761 × 10^3^	1.3511 × 10^4^	1.0191 × 10^4^	1.3792 × 10^6^	6.1287 × 10^8^	1.1925 × 10^5^	6.9965 × 10^1^
F19	7.6337 × 10^3^	2.6306 × 10^3^	8.4864 × 10^3^	1.1906 × 10^4^	1.8340 × 10^4^	1.0652 × 10^7^	6.5601 × 10^4^	5.5813
F20	4.6099 × 10^1^	5.0520 × 10^1^	9.0633 × 10^1^	6.7195 × 10^1^	5.8024 × 10^1^	4.1409 × 10^1^	5.7978 × 10^1^	1.3454 × 10^1^
F21	3.4899 × 10^1^	7.1972 × 10^1^	5.2179 × 10^1^	3.2437 × 10^1^	3.9614 × 10^1^	6.9485 × 10^1^	2.7548 × 10^1^	6.5936 × 10^1^
F22	2.3855 × 10^1^	4.6529 × 10^1^	2.1309 × 10^2^	2.6350 × 10^2^	4.3237 × 10^2^	4.3150 × 10^2^	8.2597	1.8371 × 10^1^
F23	9.8477	1.8153 × 10^1^	1.9001 × 10^1^	2.3358 × 10^1^	3.2453 × 10^1^	3.3027 × 10^1^	1.3969 × 10^1^	7.9853
F24	8.9663 × 10^1^	7.8188 × 10^1^	1.0152 × 10^2^	4.6834 × 10^1^	6.1779 × 10^1^	1.1091 × 10^2^	7.5869 × 10^1^	8.4826 × 10^1^
F25	2.6453 × 10^1^	5.8833 × 10^1^	2.6868 × 10^1^	5.9959 × 10^1^	2.4807 × 10^2^	3.0698 × 10^2^	2.8520 × 10^1^	2.4668 × 10^1^
F26	3.0883 × 10^2^	3.8933 × 10^2^	4.1253 × 10^2^	5.3401 × 10^2^	3.7620 × 10^2^	1.9171 × 10^2^	1.6743 × 10^2^	8.5319 × 10^1^
F27	2.4819 × 10^1^	2.3157 × 10^1^	4.5813 × 10^1^	5.1675 × 10^1^	5.3861 × 10^1^	5.7689 × 10^1^	6.4917	1.1351 × 10^1^
F28	1.5188 × 10^2^	1.7391 × 10^2^	1.1196 × 10^2^	2.3199 × 10^2^	1.3939 × 10^2^	1.0777 × 10^2^	9.8828 × 10^1^	1.4011 × 10^2^
F29	5.2242 × 10^1^	5.6719 × 10^1^	7.4825 × 10^1^	6.9211 × 10^1^	8.7135 × 10^1^	9.4258 × 10^1^	4.4585 × 10^1^	4.7423 × 10^1^
F30	1.4232 × 10^6^	1.0522 × 10^6^	4.9450 × 10^5^	3.5079 × 10^6^	5.7574 × 10^6^	1.3829 × 10^7^	2.0131 × 10^6^	4.2247 × 10^5^

**Table 2:** CEC 2017 test set average fitness value results with dimensions of 10.

Func.	BKAPI	BKA	PSO	GA	COA	BWO	AO	SFOA
F1	5.7144 × 10^3^	2.7818 × 10^7^	2.3374 × 10^6^	8.4230 × 10^6^	1.0105 × 10^10^	1.6695 × 10^10^	1.5082 × 10^8^	2.2207 × 10^5^
F3	3.0000 × 10^2^	1.0821 × 10^3^	3.0976 × 10^2^	5.4669 × 10^4^	1.2046 × 10^4^	1.1962 × 10^4^	4.5568 × 10^3^	3.1345 × 10^2^
F4	4.1266 × 10^2^	4.3410 × 10^2^	4.1483 × 10^2^	4.8501 × 10^2^	1.2231 × 10^3^	1.8883 × 10^3^	4.4643 × 10^2^	4.0651 × 10^2^
F5	5.1496 × 10^2^	5.3467 × 10^2^	5.3177 × 10^2^	5.8888 × 10^2^	5.8187 × 10^2^	6.2033 × 10^2^	5.3820 × 10^2^	5.4020 × 10^2^
F6	6.0008 × 10^2^	6.2945 × 10^2^	6.0824 × 10^2^	6.6285 × 10^2^	6.4253 × 10^2^	6.6256 × 10^2^	6.2424 × 10^2^	6.0373 × 10^2^
F7	7.2183 × 10^2^	7.6051 × 10^2^	7.3049 × 10^2^	8.3445 × 10^2^	8.0451 × 10^2^	8.3673 × 10^2^	7.6521 × 10^2^	7.5763 × 10^2^
F8	8.1329 × 10^2^	8.2237 × 10^2^	8.2086 × 10^2^	8.8030 × 10^2^	8.5356 × 10^2^	8.6124 × 10^2^	8.2507 × 10^2^	8.4099 × 10^2^
F9	9.0150 × 10^2^	1.2102 × 10^3^	9.3258 × 10^2^	1.0153 × 10^3^	1.4100 × 10^3^	1.9390 × 10^3^	1.1077 × 10^3^	9.2572 × 10^2^
F10	1.6796 × 10^3^	1.8682 × 10^3^	1.8948 × 10^3^	2.5509 × 10^3^	2.5722 × 10^3^	2.6004 × 10^3^	2.1646 × 10^3^	2.1071 × 10^3^
F11	1.1408 × 10^3^	1.1551 × 10^3^	1.1496 × 10^3^	7.4309 × 10^3^	2.3615 × 10^3^	1.0362 × 10^4^	1.4203 × 10^3^	1.1190 × 10^3^
F12	4.5173 × 10^5^	2.0441 × 10^5^	2.8908 × 10^5^	5.2373 × 10^6^	2.5052 × 10^8^	6.8974 × 10^8^	3.3312 × 10^6^	8.8845 × 10^3^
F13	9.7457 × 10^3^	2.9486 × 10^3^	9.3106 × 10^3^	1.6640 × 10^4^	1.5246 × 10^4^	2.2063 × 10^7^	2.2634 × 10^4^	1.4287 × 10^3^
F14	1.5448 × 10^3^	1.4879 × 10^3^	5.2661 × 10^3^	8.7232 × 10^3^	1.5219 × 10^3^	1.8836 × 10^3^	2.8204 × 10^3^	1.4326 × 10^3^
F15	2.5258 × 10^3^	1.6583 × 10^3^	6.0420 × 10^3^	1.2770 × 10^4^	5.8516 × 10^3^	9.3150 × 10^3^	9.3386 × 10^3^	1.5379 × 10^3^
F16	1.7225 × 10^3^	1.7884 × 10^3^	1.8611 × 10^3^	1.9016 × 10^3^	2.0390 × 10^3^	2.2674 × 10^3^	1.8753 × 10^3^	1.6741 × 10^3^
F17	1.7760 × 10^3^	1.7665 × 10^3^	1.7908 × 10^3^	1.7836 × 10^3^	1.8113 × 10^3^	1.8570 × 10^3^	1.7809 × 10^3^	1.7668 × 10^3^
F18	2.1646 × 10^4^	4.5652 × 10^3^	1.9866 × 10^4^	1.6269 × 10^4^	2.7169 × 10^5^	5.6907 × 10^8^	5.7860 × 10^4^	1.8861 × 10^3^
F19	6.3909 × 10^3^	2.5461 × 10^3^	9.1281 × 10^3^	1.2543 × 10^4^	1.2310 × 10^4^	6.3083 × 10^6^	4.6974 × 10^4^	1.9095 × 10^3^
F20	2.0629 × 10^3^	2.1130 × 10^3^	2.1265 × 10^3^	2.2195 × 10^3^	2.1859 × 10^3^	2.2717 × 10^3^	2.1485 × 10^3^	2.0620 × 10^3^
F21	2.3032 × 10^3^	2.2793 × 10^3^	2.3011 × 10^3^	2.3849 × 10^3^	2.3593 × 10^3^	2.3560 × 10^3^	2.3276 × 10^3^	2.2963 × 10^3^
F22	2.3006 × 10^3^	2.3218 × 10^3^	2.3363 × 10^3^	2.5283 × 10^3^	3.1260 × 10^3^	3.1485 × 10^3^	2.3212 × 10^3^	2.3015 × 10^3^
F23	2.6236 × 10^3^	2.6356 × 10^3^	2.6525 × 10^3^	2.7017 × 10^3^	2.7057 × 10^3^	2.7371 × 10^3^	2.6479 × 10^3^	2.6287 × 10^3^
F24	2.7236 × 10^3^	2.7463 × 10^3^	2.7341 × 10^3^	2.8666 × 10^3^	2.8774 × 10^3^	2.9097 × 10^3^	2.7520 × 10^3^	2.7271 × 10^3^
F25	2.9232 × 10^3^	2.9500 × 10^3^	2.9307 × 10^3^	3.0512 × 10^3^	3.4507 × 10^3^	3.9118 × 10^3^	2.9521 × 10^3^	2.9216 × 10^3^
F26	3.0634 × 10^3^	3.1413 × 10^3^	3.1233 × 10^3^	3.8855 × 10^3^	4.2061 × 10^3^	3.8658 × 10^3^	3.0937 × 10^3^	2.9269 × 10^3^
F27	3.1103 × 10^3^	3.1082 × 10^3^	3.1444 × 10^3^	3.2222 × 10^3^	3.1913 × 10^3^	3.2002 × 10^3^	3.1088 × 10^3^	3.0951 × 10^3^
F28	3.3710 × 10^3^	3.2789 × 10^3^	3.3290 × 10^3^	3.5458 × 10^3^	3.7234 × 10^3^	3.8608 × 10^3^	3.4782 × 10^3^	3.2701 × 10^3^
F29	3.2029 × 10^3^	3.2439 × 10^3^	3.2724 × 10^3^	3.3550 × 10^3^	3.3977 × 10^3^	3.5537 × 10^3^	3.2916 × 10^3^	3.2194 × 10^3^
F30	8.7553 × 10^5^	6.9174 × 10^5^	2.9351 × 10^5^	3.3497 × 10^6^	5.1961 × 10^6^	1.5161 × 10^7^	1.7869 × 10^6^	2.4944 × 10^5^

**Table 3:** CEC 2017 test set 10DIM-WILCOXON rank and test results with dimensions of 10.

Func.	BKA	PSO	GA	COA	BWO	AO	SFOA
F1	3.0198 × 10^−11^	1.2467 × 10^−2^	3.0198 × 10^−11^	3.0198 × 10^−11^	3.0198 × 10^−11^	3.0198 × 10^−11^	3.0198 × 10^−11^
F3	2.9376 × 10^−11^	2.9376 × 10^−11^	2.9376 × 10^−11^	2.9376 × 10^−11^	2.9376 × 10^−11^	2.9376 × 10^−11^	2.9376 × 10^−11^
F4	3.0058 × 10^−4^	4.8066 × 10^−4^	1.5580 × 10^−8^	3.0198 × 10^−11^	3.0198 × 10^−11^	8.8410 × 10^−7^	2.6805 × 10^−4^
F5	9.4903 × 10^−7^	3.5541 × 10^−6^	2.9953 × 10^−11^	2.9953 × 10^−11^	2.9953 × 10^−11^	1.0852 × 10^−10^	6.6430 × 10^−11^
F6	3.0198 × 10^−11^	7.3890 × 10^−11^	3.0198 × 10^−11^	3.0198 × 10^−11^	3.0198 × 10^−11^	3.0198 × 10^−11^	3.0198 × 10^−11^
F7	4.9751 × 10^−11^	9.2112 × 10^−5^	3.0198 × 10^−11^	3.0198 × 10^−11^	3.0198 × 10^−11^	3.6897 × 10^−11^	6.6955 × 10^−11^
F8	1.1683 × 10^−4^	3.0906 × 10^−3^	2.9728 × 10^−11^	2.9728 × 10^−11^	2.9728 × 10^−11^	4.0862 × 10^−6^	8.0313 × 10^−11^
F9	2.4918 × 10^−11^	9.1737 × 10^−5^	3.0497 × 10^−11^	2.4918 × 10^−11^	2.4918 × 10^−11^	2.4918 × 10^−11^	4.2850 × 10^−10^
F10	1.0762 × 10^−2^	1.3271 × 10^−2^	2.3714 × 10^−10^	6.6955 × 10^−11^	1.6132 × 10^−10^	3.3241 × 10^−6^	1.8681 × 10^−5^
F11	5.8281 × 10^−3^	2.9205 × 10^−2^	1.9567 × 10^−10^	3.0198 × 10^−11^	3.0198 × 10^−11^	8.4847 × 10^−9^	9.7051 × 10^−1^
F12	1.0314 × 10^−2^	8.1874 × 10^−1^	1.6947 × 10^−9^	3.0198 × 10^−11^	3.0198 × 10^−11^	3.4971 × 10^−9^	9.0687 × 10^−3^
F13	3.1820 × 10^−4^	9.9410 × 10^−1^	4.6371 × 10^−3^	1.0314 × 10^−2^	3.0198 × 10^−11^	8.8828 × 10^−6^	1.4643 × 10^−10^
F14	1.5797 × 10^−1^	5.5999 × 10^−7^	6.6955 × 10^−11^	1.2235 × 10^−1^	6.0103 × 10^−8^	2.4386 × 10^−9^	5.4940 × 10^−11^
F15	2.8388 × 10^−4^	5.9705 × 10^−5^	4.9979 × 10^−9^	1.1077 × 10^−6^	3.8201 × 10^−10^	3.8248 × 10^−9^	3.8201 × 10^−10^
F16	5.8281 × 10^−3^	7.6972 × 10^−4^	4.4204 × 10^−6^	2.6694 × 10^−9^	3.6897 × 10^−11^	2.7725 × 10^−5^	6.2040 × 10^−1^
F17	3.4028 × 10^−1^	8.4999 × 10^−2^	5.0120 × 10^−2^	2.1264 × 10^−4^	7.5991 × 10^−7^	3.5136 × 10^−2^	2.3985 × 10^−1^
F18	3.8052 × 10^−7^	7.2826 × 10^−1^	4.5529 × 10^−1^	7.1718 × 10^−1^	3.0198 × 10^−11^	1.2732 × 10^−2^	3.0198 × 10^−11^
F19	3.0102 × 10^−7^	2.9205 × 10^−2^	4.0329 × 10^−3^	6.6688 × 10^−3^	3.6897 × 10^−11^	2.8789 × 10^−6^	3.0198 × 10^−11^
F20	2.4327 × 10^−5^	4.6371 × 10^−3^	2.8715 × 10^−10^	3.4971 × 10^−9^	3.0198 × 10^−11^	1.4733 × 10^−7^	2.3243 × 10^−2^
F21	4.8251 × 10^−1^	3.3874 × 10^−2^	1.1737 × 10^−9^	3.8349 × 10^−6^	2.4156 × 10^−2^	4.3106 × 10^−8^	9.8834 × 10^−3^
F22	5.4620 × 10^−6^	1.1198 × 10^−1^	7.6949 × 10^−8^	3.0198 × 10^−11^	3.0198 × 10^−11^	1.3111 × 10^−8^	6.5486 × 10^−4^
F23	4.6365 × 10^−3^	1.2017 × 10^−8^	3.0179 × 10^−11^	3.0179 × 10^−11^	3.0179 × 10^−11^	5.9644 × 10^−9^	1.2730 × 10^−2^
F24	2.6069 × 10^−2^	6.3743 × 10^−3^	1.4608 × 10^−10^	3.8116 × 10^−10^	5.4511 × 10^−9^	9.7829 × 10^−5^	2.8119 × 10^−2^
F25	2.5188 × 10^−1^	2.2823 × 10^−1^	3.0198 × 10^−11^	3.0198 × 10^−11^	3.0198 × 10^−11^	1.5291 × 10^−5^	8.7663 × 10^−1^
F26	7.1715 × 10^−1^	6.7345 × 10^−1^	2.9068 × 10^−9^	2.5950 × 10^−10^	3.8059 × 10^−9^	1.2719 × 10^−2^	3.7755 × 10^−2^
F27	6.7349 × 10^−1^	1.0184 × 10^−5^	2.6083 × 10^−10^	3.8229 × 10^−9^	2.4373 × 10^−9^	2.2358 × 10^−2^	2.3160 × 10^−6^
F28	2.3065 × 10^−2^	2.2964 × 10^−1^	9.7882 × 10^−3^	1.9388 × 10^−9^	4.6986 × 10^−11^	3.7430 × 10^−5^	3.8025 × 10^−3^
F29	2.1566 × 10^−3^	6.2828 × 10^−6^	3.1967 × 10^−9^	6.1210 × 10^−10^	4.5043 × 10^−11^	4.1127 × 10^−7^	4.8413 × 10^−2^
F30	7.2825 × 10^−1^	5.3948 × 10^−1^	1.6809 × 10^−4^	5.1819 × 10^−7^	2.2261 × 10^−9^	2.0520 × 10^−3^	2.5300 × 10^−4^

**Table 4:** Standard deviation and average fitness value results of the CEC 2022 test set.

Func.	Type	BKAPI	BKA	PSO	GA	COA	BWO	AO	SFOA
F1	std	0.00	1377.83	5.35	14,486.18	1750.42	40,282.57	2857.41	18.73
	avg	300.00	710.61	301.60	36,199.35	8169.68	41,983.55	6257.44	316.96
F2	std	22.10	28.72	27.25	26.88	683.43	918.34	53.28	2.12
	avg	416.88	417.01	422.02	470.32	1426.23	2387.02	460.50	406.93
F3	std	0.44	10.59	6.00	13.92	10.10	8.34	8.52	2.50
	avg	600.19	632.08	605.22	664.35	644.91	663.13	624.78	603.93
F4	std	6.39	7.05	9.79	14.36	7.83	7.02	9.28	9.51
	avg	814.36	819.65	821.36	879.23	848.49	856.05	826.26	841.56
F5	std	1.48	115.74	41.83	32.73	158.33	172.12	145.76	75.53
	avg	900.78	1115.52	922.27	948.07	1383.78	1785.92	1129.73	951.38
F6	std	2451.28	1525.48	2164.75	3962.44	5,256,162.17	983,998,623	153,581.98	1116.90
	avg	5129.50	3110.97	3638.71	5141.80	2,669,099.83	1,214,747,876.1	130,314.12	2166.58
F7	std	5.67	28.93	17.20	30.20	14.77	19.88	20.75	7.28
	avg	2021.87	2052.37	2036.44	2099.17	2088.07	2132.00	2063.63	2034.02
F8	std	5.33	36.48	51.46	42.72	5.20	25.72	5.23	3.74
	avg	2219.80	2239.15	2249.11	2264.19	2232.27	2273.82	2232.02	2227.02
F9	std	24.80	35.30	44.65	53.86	35.11	43.29	36.44	0.00
	avg	2535.82	2544.67	2545.49	2691.61	2731.18	2787.40	2632.55	2529.28
F10	std	61.34	87.38	102.63	364.52	187.92	156.79	65.17	45.79
	avg	2556.42	2571.02	2590.35	2698.17	2722.48	2749.22	2569.72	2518.29
F11	std	33.47	256.42	57.02	474.68	488.69	477.34	75.51	99.96
	avg	2898.77	2866.19	2912.42	3595.09	3926.81	3565.78	2774.76	2712.29
F12	std	13.24	15.90	11.97	59.66	44.06	80.73	6.56	1.65
	avg	2872.03	2872.60	2875.13	2984.69	2961.55	2985.14	2871.84	2861.14

**Table 5:** CEC 20122 test set 10DIM-WILCOXON rank and test results with dimensions of 10.

Func.	BKA	PSO	GA	COA	BWO	AO	SFOA
F1	2.8358 × 10^−11^	2.8358 × 10^−11^	2.8358 × 10^−11^	2.8358 × 10^−11^	2.8358 × 10^−11^	2.8358 × 10^−11^	2.8358 × 10^−11^
F2	1.8264 × 10^−2^	6.5526 × 10^−1^	1.5130 × 10^−8^	2.9008 × 10^−11^	2.9008 × 10^−11^	2.1422 × 10^−7^	4.5489 × 10^−1^
F3	3.0198 × 10^−11^	4.5725 × 10^−9^	3.0198 × 10^−11^	3.0198 × 10^−11^	3.0198 × 10^−11^	3.0198 × 10^−11^	5.4940 × 10^−11^
F4	7.6868 × 10^−4^	2.7447 × 10^−3^	3.0047 × 10^−11^	3.3217 × 10^−11^	3.0047 × 10^−11^	9.5068 × 10^−7^	1.7686 × 10^−10^
F5	2.9247 × 10^−11^	2.1075 × 10^−6^	2.9247 × 10^−11^	2.9247 × 10^−11^	2.9247 × 10^−11^	2.9247 × 10^−11^	4.3648 × 10^−11^
F6	1.1142 × 10^−3^	1.8367 × 10^−2^	6.3087 × 10^−1^	2.0152 × 10^−8^	3.0198 × 10^−11^	2.6098 × 10^−10^	1.6979 × 10^−8^
F7	2.1947 × 10^−8^	2.9589 × 10^−5^	3.0198 × 10^−11^	3.0198 × 10^−11^	3.0198 × 10^−11^	3.6897 × 10^−11^	2.6694 × 10^−9^
F8	1.4733 × 10^−7^	6.7868 × 10^−2^	3.0198 × 10^−11^	4.0771 × 10^−11^	3.0198 × 10^−11^	3.0198 × 10^−11^	1.0104 × 10^−8^
F9	4.3711 × 10^−9^	5.9482 × 10^−2^	3.9348 × 10^−12^	3.1578 × 10^−12^	3.1578 × 10^−12^	3.3715 × 10^−11^	2.4772 × 10^−8^
F10	3.5136 × 10^−2^	4.5143 × 10^−2^	6.6688 × 10^−3^	1.1674 × 10^−5^	1.8608 × 10^−6^	3.0339 × 10^−3^	8.0727 × 10^−1^
F11	7.8782 × 10^−2^	2.1155 × 10^−9^	4.5329 × 10^−7^	8.7486 × 10^−12^	8.7486 × 10^−12^	1.8630 × 10^−7^	5.7769 × 10^−8^
F12	4.3760 × 10^−1^	1.6271 × 10^−2^	3.6782 × 10^−11^	1.2014 × 10^−10^	1.2014 × 10^−10^	1.7142 × 10^−1^	4.1880 × 10^−10^

**Table 6:** Welding beam project index statistics.

Algorithm	Optimal Value	Worst Value	Standard Deviation	Average Value	Median Value	Average Time
BKAPI	1.6702	1.7817	0.0537	1.6883	1.6751	2.9511
BKA	1.6709	1.7012	0.6598	1.6781	1.6739	0.6954
PSO	1.6728	2.5874	0.2812	1.8164	1.7052	0.3050
GA	1.8322	2.5977	0.2272	2.0912	2.0320	0.5071
COA	1.8455	2.2295	0.1180	2.1363	2.1934	0.7834
BWO	2.3636	4.0189	0.6031	3.2940	3.4059	2.8143
AO	1.8143	2.4377	0.2363	2.0917	2.0713	0.6454
SFOA	1.6706	1.6742	0.0711	1.6722	1.6719	0.2899

**Table 7:** The welded beam design problem’s best outcomes from the various algorithms.

Algorithm	Optimal Values for Variables				Optimal Cost
	*h*	*l*	*t*	*b*	
BKAPI	0.1988	3.3374	9.1920	0.1988	1.6702
BKA	0.1983	3.3487	9.1920	0.1988	1.6709
PSO	0.1985	3.3436	9.2032	0.1989	1.6728
GA	0.1592	4.6256	9.0043	0.2110	1.8322
COA	0.1473	5.1905	9.4258	0.1978	1.8455
BWO	0.3657	2.4001	6.6664	0.3819	2.3636
AO	0.1669	4.2151	8.9001	0.2160	1.8143
SFOA	0.1988	3.3390	9.1942	0.1988	1.6706

**Table 8:** Himmelblau function’s best outcomes from the various algorithms.

Algorithm	Optimal Values for Variables					Optimal Cost
	$x_{1}$	$x_{2}$	$x_{3}$	$x_{4}$	$x_{5}$	
BKAPI	78.0000	33.0000	29.9953	45.0000	36.7758	−30,665
BKA	78.0000	33.0000	29.9953	45.0000	36.7759	−30,665
PSO	78.0000	33.0000	29.9953	45.0000	36.7758	−30,665
GA	80.8815	35.6805	32.0422	37.5918	34.4031	−29,802
COA	78.0000	33.0000	30.0108	44.9387	36.7616	−30,623
BWO	78.0000	33.0000	31.7258	42.1734	35.1803	−30,266
AO	78.5773	33.2768	30.4269	44.2749	35.9693	−30,544
SFOA	78.0000	33.0000	29.9953	45.0000	36.7757	−30,665

**Table 9:** Himmelblau function optimization index statistics.

Algorithm	Optimal Value	Worse Value	Standard Deviation	Average Value	Median Value	Average Time
BKAPI	−30,665	−30,186	151	−30,617	−30,665	3.68
BKA	−30,665	−30,186	150	−30,615	−30,662	0.68
PSO	−30,665	−30,662	1	−30,665	−30,665	0.29
GA	−29,802	−28,895	259	−29,445	−29,421	0.53
COA	−30,623	−29,690	323	−30,160	−30,142	0.75
BWO	−30,266	−29,594	187	−30,028	−30,069	3.91
AO	−30,544	−29,753	235	−30,240	−30,258	0.69
SFOA	−30,665	−30,424	76	−30,640	−30,663	0.31

## Data Availability

The data that support the findings of this study are available from the corresponding author upon request. There are no restrictions on data availability.

## Abbreviations

The following abbreviations are used in this manuscript:

BKA	Black-Winged Kite Algorithm
PSO	Particle Swarm Optimization
BKAPI	Hybrid Black-Winged Kite Algorithm with Particle Swarm Optimization
VLP	Visible Light Positioning
